# Brain-Sparing *Sympathofacilitators* Mitigate Obesity without Adverse Cardiovascular Effects

**DOI:** 10.1016/j.cmet.2020.04.013

**Published:** 2020-06-02

**Authors:** Inês Mahú, Andreia Barateiro, Eva Rial-Pensado, Noelia Martinéz-Sánchez, Sandra H. Vaz, Pedro M.S.D. Cal, Benjamin Jenkins, Tiago Rodrigues, Carlos Cordeiro, Miguel F. Costa, Raquel Mendes, Elsa Seixas, Mafalda M.A. Pereira, Nadiya Kubasova, Vitka Gres, Imogen Morris, Carolina Temporão, Marta Olivares, Yolanda Sanz, Albert Koulman, Francisco Corzana, Ana M. Sebastião, Miguel López, Gonçalo J.L. Bernardes, Ana I. Domingos

**Affiliations:** 1 Department of Physiology, Anatomy and Genetics, University of Oxford, Parks Road, Oxford OX1 3PT, UK; 2 Obesity Laboratory, Instituto Gulbenkian de Ciência, Oeiras 2780-156, Portugal; 3 Neuron Glia Biology in Health and Disease, Research Institute for Medicines (iMed.ULisboa), Faculty of Pharmacy, Universidade de Lisboa, Lisbon 1649-028, Portugal; 4 NeurObesity Group, Department of Physiology, CIMUS, University of Santiago de Compostela, Instituto de Investigación Sanitaria, Santiago de Compostela, A Coruña 15782, Spain; 5 Instituto de Medicina Molecular, João Lobo Antunes, Faculdade de Medicina, Universidade de Lisboa, Av. Prof., Egas Moniz, Lisbon 1649-028, Portugal; 6 Instituto de Farmacologia e Neurociências, Faculdade de Medicina, Universidade de Lisboa, Av. Prof. Egas Moniz, Lisboa 1649-028, Portugal; 7 NIHR BRC Core Metabolomics and Lipidomics Laboratory, Wellcome Trust, MRL Institute of Metabolic Science, University of Cambridge, Pathology building Level 4, Addenbrooke’s Hospital, Cambridge CB2 0QQ, UK; 8 Laboratório de FT-ICR e Espectrometria de Massa Estrutural, Faculdade de Ciências da Universidade de Lisboa, Lisbon 1749-016, Portugal; 9 Department of Bioengineering, Instituto Superior Técnico, Universidade de Lisboa, Lisbon 1049-001, Portugal; 10 Microbial Ecology, Nutrition & Health Research Unit, Institute of Agrochemistry and Food Technology, National Research Council, Valencia (IATA-CSIC), Catedratico Agustin Escardino 7, 46980, Paterna, Valencia, Spain; 11 Departamento de Química, Universidad de La Rioja, Centro de Investigación en Síntesis Química, 26006 Logroño, Spain; 12 Department of Chemistry, University of Cambridge, Lensfield Road, Cambridge CB2 1EW, UK; 13 Howard Hughes Medical Institute, IGC, Oeiras, Portugal

**Keywords:** obesity, sympathetic-nervous-system, *sympathofacilitators*, sympathomimetics, amphetamine, lipolysis, thermogenesis, heat dissipation, thermoregulation, tachycardia

## Abstract

Anti-obesity drugs in the amphetamine (AMPH) class act in the brain to reduce appetite and increase locomotion. They are also characterized by adverse cardiovascular effects with origin that, despite absence of any *in vivo* evidence, is attributed to a direct sympathomimetic action in the heart. Here, we show that the cardiac side effects of AMPH originate from the brain and can be circumvented by PEGylation (PEGyAMPH) to exclude its central action. PEGyAMPH does not enter the brain and facilitates SNS activity via theβ_2_-adrenoceptor, protecting mice against obesity by increasing lipolysis and thermogenesis, coupled to higher heat dissipation, which acts as an energy *sink* to increase energy expenditure without altering food intake or locomotor activity. Thus, we provide proof-of-principle for a novel class of exclusively peripheral anti-obesity *sympathofacilitators* that are devoid of any cardiovascular and brain-related side effects.

## Highlights

• PEGylated amphetamine (PEGyAMPH) does not enter the brain, nor has behavioral effects
• PEGyAMPH promotes weight loss, by coupling thermogenesis to heat dissipation
• PEGyAMPH is a *sympathofacilitator*, requiring β_2_-adrenoceptor function
• PEGyAMPH is cardioprotective, unless directly delivered into the brain

## Graphical Abstract

**Figure:**
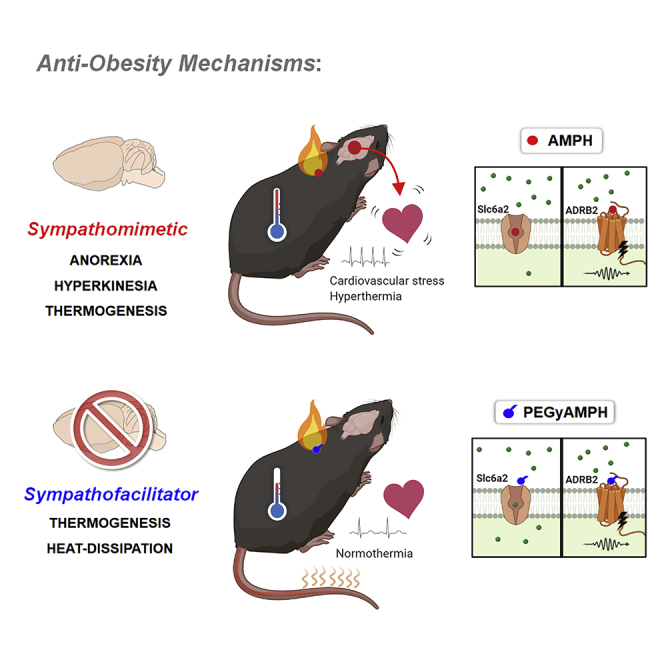

## Teaser

PEGyAMPH is a brain-sparing modified amphetamine that is a *cardioneutral* anti-obesity drug. Unlike amphetamines, which act in the brain to increase satiety and locomotion, PEGyAMPH couples thermogenesis to heat dissipation, activating a whole-body energy sink by simultaneously promoting vasodilation and facilitating the activity of sympathetic neurons.

## Context and Significance

**Amphetamine-like drugs have anti-obesity action, which is associated with increased satiety and locomotion, but are known to cause cardiac side effects. Mahú et al. found that these side effects originate centrally and created a brain-sparing drug by chemically modifying amphetamine through PEGylation (PEGyAMPH). This new molecule promotes weight loss via molecular and physiological mechanisms, which are unrelated to those of centrally acting amphetamines. PEGyAMPH couples heat production to its simultaneous dissipation to act as a whole-body energy sink. It acts via β_**2**_-adrenoceptors to promote vasodilation, while facilitating the activity of fat burning peripheral neurons. These findings provide proof-of principle for the development of anti-obesity drugs that act peripherally, while circumventing the brain.**

## Introduction

Anti-obesity drugs in the amphetamine (AMPH) class, such as FDA-approved phentermine, are highly efficacious therapeutic compounds approved for common obesity (Cooke and Bloom, 2006, Melnikova and Wages, 2006). The potent anti-obesity action of this class of drugs is reportedly mediated by a stimulant action in the brain that suppresses appetite and promotes hyperkinesia (Cooke and Bloom, 2006, Heal et al., 2013, Melnikova and Wages, 2006). Although their anti-obesity effects are unparalleled, these drugs are not only addictive, but also drive cardiovascular side effects, such as tachycardia and hypertension. It is so far unclear whether these side effects originate peripherally or centrally in the brain. Central action is a viable possibility, as the brain robustly controls heart rate and vascular capacitance in response to multiple internal and external stimuli (Malpas, 2010). However, despite the lack of direct experimental evidence, the peripheral model has prevailed, wholly on empirical grounds. Specifically, no direct evidence exists regarding the *in vivo* origin of any cardiovascular side effects or whether a *cardioneutral* anti-obesity effect could result if AMPH is excluded from the brain.

All AMPHs are coined as *indirect sympathomimetics* because they block monoamine transporters, thus increasing catecholamine availability (Cooke and Bloom, 2006, Heal et al., 2013, Melnikova and Wages, 2006). Recent evidence demonstrates that the genetic loss of function of norepinephrine (NE) transporter (*Slc6a2*) outside the brain promotes weight loss, without any changes in food intake (FI) or locomotor activity (LA) (Pirzgalska et al., 2017). As such, we hypothesized that preventing access of *sympathomimetic* drugs to the brain would be sufficient to promote weight loss, independent of behavior. To test this hypothesis, we chemically modified AMPH by PEGylation to increase its hydrodynamic radius and prevent its access to the brain (Pereira et al., 2017). PEGyAMPH does not cross the blood-brain barrier (BBB), yet retains the capacity to facilitate activation of sympathetic neurons and to increase peripheral NE availability in adipose tissues. Orthogonal to our initial hypothesis, we uncovered that this effect is mediated by engagement with the β_2_-adrenoceptor (ADRB2), a well-known mediator of vasodilation and smooth muscle relaxation (Chruscinski et al., 1999, Ernande et al., 2016). We found that unlike AMPH, PEGyAMPH does not block Slc6a2 and it is devoid of cardiovascular effects, which re-emerge if directly delivered to the brain. PEGyAMPH has an anti-obesity size effect similar to that of AMPH, yet without suppression of FI or increased LA. Its anti-obesity effect is attributable to elevated lipolysis and lipid utilization, and increased thermogenesis coupled with higher heat dissipation, which combinedly contribute to *sink* energy while overriding caloric intake (Jung et al., 1979, Kasza et al., 2019, Schwartz et al., 1983, Wang, 1924, Warner et al., 2013).

## Results

### The Anti-obesity Effect of Amphetamines Requires an Intact SNS

**Figure 1:**
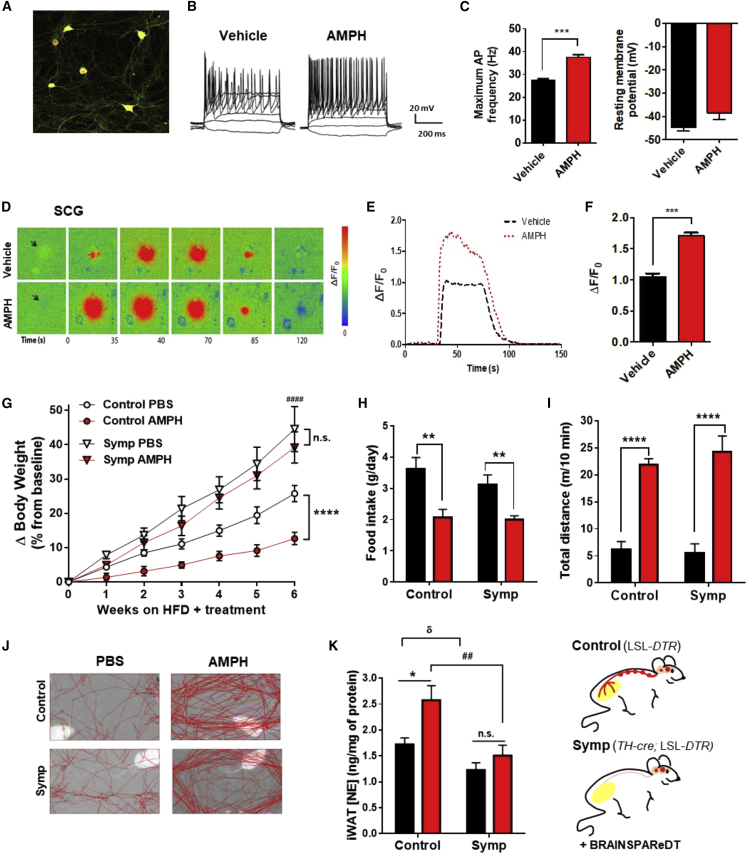
Amphetamine (AMPH) Facilitates SNS Activation, Which Is Required for the Anti-obesity Effect, Independently of Hypophagia and Hyperkinesia (A) Cultured GCaMP3^+^ SCG neurons immuno-labeled for tyrosine hydroxylase (TH). (B) Representative traces of changes in membrane potential and action potential (AP) evoked under current-clamp mode in Vehicle and AMPH-treated SCG neurons. (C) Maximum AP firing frequency and resting membrane potential. (D) Sequence of representative pseudocolor images showing calcium levels ([Ca^2+^]_i_) in GCaMP3^+^ neurons after stimulation with ACh. Changes in fluorescence (ΔF) are expressed as ΔF/F_0_ = [(F_post_ − F_rest_)/F_rest_] and represented in pseudocolor scale. (E) Representative ACh-induced [Ca^2+^]_i_ response tracings. (F) Amplitude of ACh-induced [Ca^2+^]_i_ transients (n = 8; statistics done using one-way ANOVA, followed by Bonferroni correction). (G) Change in body weight (ΔBW) of control and regionally Symp mice during HFD exposure plus treatment with PBS or AMPH (120 μmol/kg of BW, daily i.p. - n = 10–13. Statistics done using two-way ANOVA). (H) Daily food intake during HFD exposure and respective treatment. (I) Representative tracking of the LA. (J) Total distance traveled in 10 min. (K) NE content in iWAT. (n = 6–12; statistics done using unpaired Student’s t test, with Holm-Sidak correction method). ^∗,δ,#^p < 0.05; ^∗^PBS versus AMPH; ^δ^control+PBS versus Symp+PBS; ^#^control+AMPH versus Symp+AMPH. Data presented as mean ± SEM. See also Figure S1.

Despite being classified as *sympathomimetic*, to the best of our knowledge, there are no literature reports on the ability of AMPH to directly activate sympathetic neurons. To bridge this literature gap, we utilized both electrophysiology and intracellular calcium ([Ca^2+^]_i_) imaging to probe the effects of AMPH on the excitability of neurons isolated from superior cervical ganglia (SCG). By recording firing patterns of sympathetic neurons from C57BL/6 mice through whole-cell patch-clamp under current-clamp mode (Figures 1A and 1B), we observed that AMPH significantly increases the maximum firing frequency (Figure 1C, left panel), without changing resting membrane potential (Figure 1C, right panel). In parallel, we also used dissociated cultures of *TH-cre;*CAG-LSL*-GCaMP3* (GCaMP3^+^) reporter mice to perform [Ca^2+^]_i_ imaging. Local application of acetylcholine (ACh), a physiologic SNS activator, leads to an [Ca^2+^]_i_ increase in GCaMP3^+^ neurons, which then results in a significantly higher response upon treatment with AMPH (Figures 1D–1F). Hence, these results confirm that AMPH treatment increases the intrinsic excitability of sympathetic neurons.

Next, to investigate whether the increase in SNS tone is required for the anti-obesity effect of AMPH, we subjected LSL-DTR (control) and sympathectomized *TH-cre*; LSL-DTR mice (Symp; Pereira et al., 2017; Figure S1A) to an obesogenic high-fat diet (HFD) plus treatment with AMPH (120 μmol/kg of body weight [BW]) or with phosphate-buffered saline (PBS) as control, via daily intraperitoneal (i.p.) injections, for a total of 6 weeks. As expected, AMPH treatment protects control mice from diet-induced obesity (DIO) (Figures 1G and S1B), and, as previously reported (Pereira et al., 2017), Symp mice become extremely prone to DIO (Figure 1G). Surprisingly, both cohorts of Symp mice had similar rate of BW-gain upon HFD exposure, regardless of treatment, which led to an approximately 40% increase after just 6 weeks (Figures 1G and S1B). This happened independent of behavioral changes (Figures 1H–1J), as, upon treatment, both control and Symp groups showed great reduction in FI (Figure 1H) and increase in LA (Figures 1I and 1J). Thus, we theorized that underlying this phenotype was the reduction in sympathetic output, which would lead to a depression of adrenergic-stimulated lipolysis (Caron et al., 2018, Pereira et al., 2017, Schwartz et al., 1983). To assess this, we quantified both the NE content of the inguinal white adipose tissue (iWAT) and the plasma levels of lipolysis markers after AMPH treatment. Indeed, Symp mice had a significantly dampened SNS response to AMPH (Figure 1K), which was accompanied by a reduction in stimulated lipolysis (Figure S1C). Combined, these results strongly support that the *sympathomimetic* activity of AMPH is required for its protection against weight gain. More importantly, the reduced FI and increased LA observed upon AMPH treatment are ineffective at reducing the rate of BW-gain in the absence of an intact SNS (Bray, 1991, Spraul et al., 1993).

### PEGylation of AMPH Prevents Its Access to the Brain and Its Behavioral Effects

**Figure 2:**
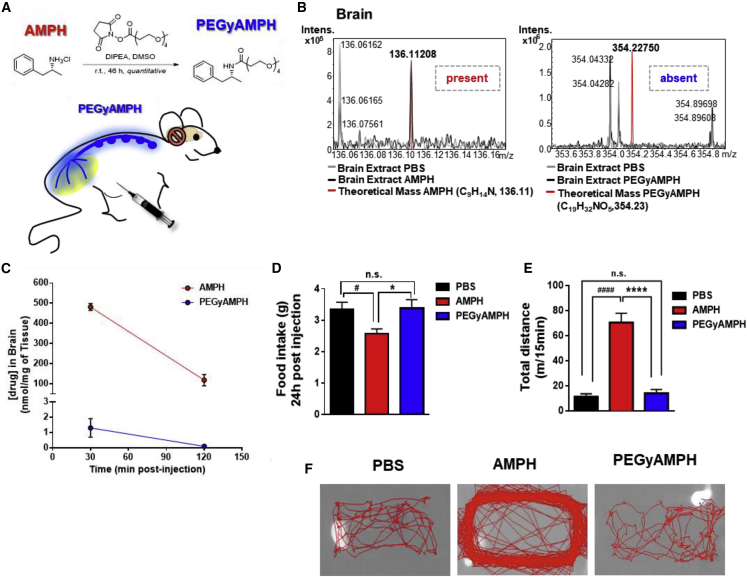
Pegylated Amphetamine (PEGyAMPH) Does Not Enter the Brain and Does Not Induce Hypophagia or Hyperkinesia (A) Representative scheme of the AMPH’s PEGylation method to produce PEGyAMPH. (B) Representative FT-ICR mass spectra of brain extracts from C57BL/6 mice 30 min post-injection with AMPH or PEGyAMPH (120 μmol/kg of BW, i.p.). (C) Brain levels of AMPH and PEGyAMPH. (D) 24 h FI post-injection with PBS, AMPH, or PEGyAMPH (normal diet). (E) Total distance traveled in 15 min. (F) Representative tracking of LA (n = 4–10; statistics done using unpaired Student’s t test, with Holm-Sidak correction method). ^∗,#^p < 0.05; ^∗^PBS versus PEGyAMPH; ^#^PBS versus AMPH. Data presented as mean ± SEM. See also Figure S2.

The BBB is generally impervious to large molecules; thus, we resorted to chemical modification of AMPH by PEGylation (Pereira et al., 2017, see STAR Methods) to increase the hydrodynamic radius size here, named as PEGyAMPH (Figure 2A). To assess the success of this modification, we injected adult C57BL/6 mice with AMPH or PEGyAMPH (120 μmol/kg of BW for both drugs, i.p.) and collected brains after 30 min and 2 h. The 30-min brain extracts were then analyzed by Fourier-transform ion cyclotron resonance (FT-ICR) mass spectrometry to detect the presence of either molecule. Given its high resolution, one can identify the compound with errors lower than 1.5 ppm, and only in the group treated with AMPH was the drug detectable (Figure 2B). Additionally, we processed brain tissues from both time points by liquid chromatography with mass spectrometry (LCMS) detection (quantitative). Possibly due to minimal penetration in areas where the BBB is not complete, we found a negligible quantity of PEGyAMPH 30 min after i.p. administration, which then became completely undetectable 2 h post-injection (Figure 2C). To consolidate our results, we then probed behavioral alterations in free-moving mice after i.p. administration of both drugs. According to our previous results, AMPH treatment consistently suppresses FI (Figure 2D) and increases LA (Figures 2E and 2F). Importantly, no changes in behavior were observed in PEGyAMPH-injected mice relative to those of the control animals (Figures 2D–2F).Hence, we could conclude that PEGylation of AMPH (PEGyAMPH) successfully restrains its brain action.

### PEGyAMPH Facilitates SNS Activation, via ADRB2 Signaling

**Figure 3:**
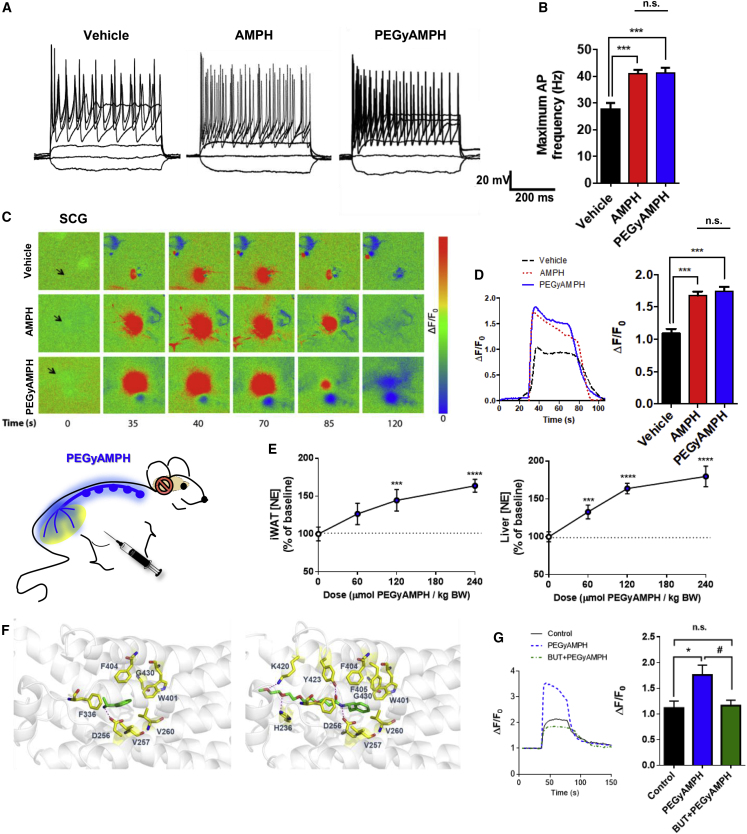
PEGyAMPH Facilitates SNS Activation via ADRB2 Signaling (A) Representative traces of changes in membrane potential and AP evoked under current-clamp mode in Vehicle, AMPH and PEGyAMPH-treated SCG neurons. (B) Maximum AP firing frequency. (C) Sequence of representative pseudocolor images of [Ca^2+^]_i_ changes after stimulation with ACh. (D) Representative ACh-induced [Ca^2+^]_i_ response tracings in Vehicle, AMPH and PEGyAMPH-treated GCaMP3^+^ neurons (left), and Amplitude of ACh-induced [Ca^2+^]_i_ transients (right). (n = 3-4; statistics done using one-way ANOVA followed by Bonferroni correction). (E) Increase in NE content of iWAT (left) and liver (right) of C57BL/6 mice post-treatment with PEGyAMPH (60, 120, or 240 μmol/kg of BW, i.p. injections). (n = 8–12; statistics done using unpaired Student’s t test, with Holm-Sidak correction method). (F) 3D structure of ADRB2 in complex with AMPH and PEGyAMPH. Left: Minimized structure for ADRB2-AMPH complex, and Right: Minimized structure for ADRB2-PEGyAMPH complex, both showing the most relevant interactions between ligand and receptor. ADBR2 is represented as white ribbons and the carbon atoms of the residues of this receptor that are interacting with the ligands are in yellow. The carbon atoms of the ligands are in green. (G) Representative ACh-induced [Ca^2+^]_i_ response (left), and Amplitude of ACh-induced [Ca^2+^]_i_ transients in GCaMP3^+^ neurons after pharmacological treatment with PEGyAMPH, in the absence or presence of butoxamine (BUT) (right). (n = 3–4; statistics done using one-way ANOVA followed by Bonferroni correction). ^∗,#^p < 0.05; ^∗^PBS versus PEGyAMPH; ^#^PEGyAMPH versus PEGyAMPH+BUT. Data presented as mean ± SEM. See also Figure S3.

To investigate PEGyAMPH’s biologic activity, we again treated SCG neurons with either AMPH or PEGyAMPH and recorded the firing patterns, using whole-cell patch-clamp (Figures 3A, 3B, and S3A–S3C). The maximum firing frequency of PEGyAMPH-treated SNS neurons increased relative to that of controls (Figure 3B), like AMPH, without changes in resting membrane potential (Figure S3A). Moreover, we found a significant increase in the AP firing threshold and in the Δ depolarization for AP firing that was only detected between Vehicle and PEGyAMPH (Figures S3B and S3C). The ACh-induced [Ca^2+^]_i_ response of GCaMP3^+^ neurons incubated with PEGyAMPH was similar to that observed in AMPH-treated neurons (Figures 3C–3E). Thus, our results confirm that PEGyAMPH, like AMPH, increases the intrinsic excitability of sympathetic neurons. To further assess whether PEGyAMPH also had the capacity to elevate the SNS tone *in vivo*, we conducted a dose response curve in C57BL/6 mice, and found the NE content of metabolic tissues to be increased in a dose-dependent manner (Figure 3E, left: iWAT; right: Liver).

We then probed the effects of PEGylation on the drug’s pharmacologic properties. AMPH accumulates in the brain and has a circulating half-life of ∼20 min in mice (Riffee et al., 1978). Our pharmacokinetic analysis revealed that PEGyAMPH has shorter plasma half-life (Figure S2A, 0.2 h versus 0.36 h, respectively), it is more quickly metabolized in the liver (Figure S2B), and, as expected, is much less excreted via urine (Figure 2C) (Harris and Chess, 2003). Additionally, to explore the functional properties of PEGyAMPH, we started by testing its capacity to bind Slc6a2 *in vitro*, as this is reported to be a major target of AMPH (Heal et al., 2013) and found a marked difference in the binding to this target: while AMPH displaces ∼80% of the radioligand at 50 μM, its PEGylated counterpart shows no activity (Figure S2D). This suggests that although the *sympathomimetic* effects are similar, the pharmacology of the two drugs differs. As such, we then evaluated the effect of replacing the NH_3_^+^ of AMPH by an amide group, in other potential interactions of PEGyAMPH with adrenoceptors for which there are available X-ray structures. For this, we conducted docking calculations for both AMPH and PEGyAMPH with either β_1_-adrenoceptor (ADRB1) or ADRB2 (Figures 3F and S3D, respectively). According to our calculations, the NH_3_^+^ group of AMPH is engaged in a hydrogen bond with the side chain of Asp256 of the ADRB2 (Figure 3F, left panel, see STAR Methods for details). In addition, the methyl group of the drug is involved in a CH-π interaction with Phe336 of the receptor, and its aromatic ring establishes hydrophobic contacts with several residues of ADRB2. The complex between PEGyAMPH and ADRB2 is stabilized by several hydrogen bonds. Of note, a prevalent one is formed between the NH group of PEGyAMPH and the side chain of Asp256 of the receptor, while an additional one involves the carbonyl group of the PEGylated compound and Tyr423 of ADBR2. Furthermore, the PEG chain is also involved in two other hydrogen bonds with the side chains of His236 and Lys240 of the ADRB2 (Figure 3F, right panel). As such, the replacement of the NH_3_^+^ by an amide group does not significantly disturb the interactions with the adrenoceptors tested, which is aligned with PEGyAMPH’s ability to modulate SNS tone to metabolic tissues (Figures 3E, S3E, and S3F).

To experimentally probe the effect of specific engagement of the ADRB2 by PEGyAMPH on its *sympathofacilitator* properties, we repeated the ACh-induced [Ca^2+^]_I_ response assays and found that butoxamine (BUT), a selective ADRB2 antagonist (Gabanyi et al., 2016), blocked PEGyAMPH’s capacity to amplify neuronal activation (Figure 3G). Hence, PEGylation of AMPH changed its pharmacology, but it did not reduce the *sympathofacilitator* activity, which relies on ADRB2 engagement.

### PEGyAMPH Does Not Affect Cardiovascular Function in Mice, Unless It Is Centrally Delivered

**Figure 4:**
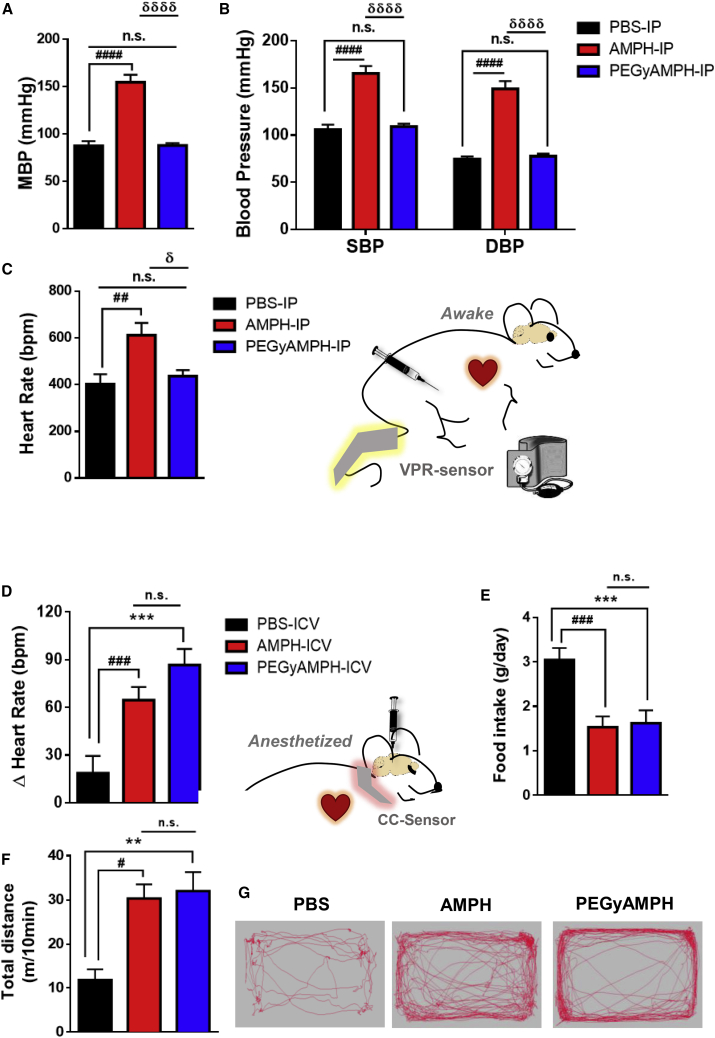
PEGyAMPH, Unlike AMPH, Does Not Affect Cardiovascular Function, Unless Delivered Centrally (A) Mean blood pressure (MBP). (B) Systolic blood pressure (SBP) and diastolic blood pressure (DBP). (C) Heart rate of C57BL/6 mice, recorded post-injection with PBS, AMPH, or PEGyAMPH (120 μmol/kg of BW for both drugs, i.p.) using a non-invasive Volume Pressure Recording (VPR) tail-cuff system. (D–G) Measurements taken post-i.c.v. injection of PBS, AMPH, or PEGyAMPH (60 nmol, bolus, per animal). (D) Change in heart rate recorded using a CollarClip Sensor (CC-Sensor) for pulse oximetry. (E) 24-h FI of i.c.v.-injected mice. (F) Total distance traveled in 10 min. (G) Representative trackings post-i.c.v. (n = 8–12; statistics done using unpaired Student’s t test, with Holm-Sidak correction method). ^∗,#,δ^p < 0.05; ^∗^PBS versus PEGyAMPH; ^#^PBS versus AMPH; ^δ^PEGyAMPH versus AMPH. Data presented as mean ± SEM. See also Figure S4.

The anti-obesity effects of AMPH-like compounds are proposed to be driven by their modulation of behavior, yet these drugs are coined *sympathomimetics* (Heal et al., 2013) in reference to their well-known cardiovascular side effects. Yet, it is so far unclear whether these originate peripherally by direct activation of the SNS by AMPH or centrally by a brain-dependent action (Heal et al., 2013). Surprisingly, we found that the peripherally acting drug did not cause elevation of blood pressure (Figures 4A and 4B) or heart rate (Figures 4C and S4A) upon i.p. administration. Concomitantly, we also detected less accumulation of the drug (Figure S4B) and of NE in the hearts (Figure S4C) of animals treated with PEGyAMPH compared with those treated with AMPH. Hence, a central action seems to be a viable possibility (Malpas, 2010). To test this, we probed the effect of central administration of both drugs by intracerebroventricular (i.c.v.) injection (bolus of 60 nmol). PEGyAMPH had equivalent anorexigenic effect (Figure 4E) and capacity to increase LA (Figures 4F and 4G) to that of AMPH. Importantly, we confirmed that i.c.v. injections were sufficient to induce excitatory effects on the cardiorespiratory system (Figures 4D and S4D). Combined, our results suggest that the well-described cardiovascular stress induced by *sympathomimetic* drugs is driven by their central action on the brain.

### PEGyAMPH Protects Mice from Obesity by Elevating EE, without Affecting Feeding Behavior or Locomotion

**Figure 5:**
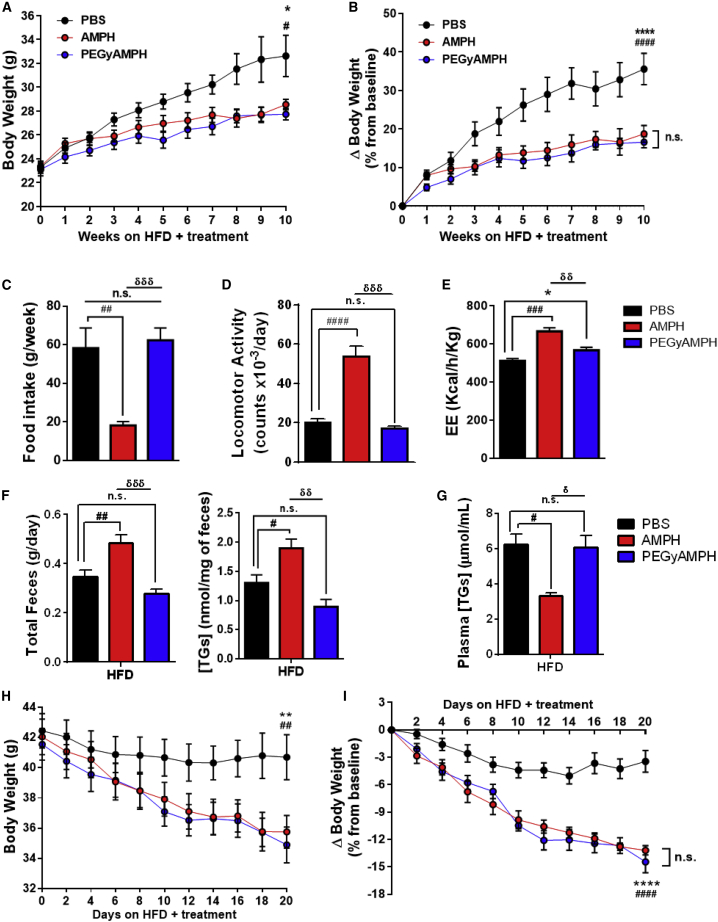
PEGyAMPH Protects Mice from DIO and Increases EE without Affecting Food Intake (A and B) BW (A) and ΔBW (B) of C57BL/6 mice during HFD exposure and treatment with PBS, AMPH, or PEGyAMPH (120 μmol/kg of BW for both drugs, daily i.p.). (C) Average FI.**(**D) Daily LA, quantified in beam-break counts. (E) EE, normalized to total BW. (F) Daily fecal output (left) and fecal TGs content (right). (G) Plasma TGs levels. (H and I) BW (H) and ΔBW (I) of DIO mice during treatment. (n = 8–15; statistics done using two-way ANOVA for the BW measurement over time, and using unpaired Student’s t tests, with Holm-Sidak correction method, for the other assays). ^∗,#,δ^p < 0.05; ^∗^PBS versus PEGyAMPH; ^#^PBS versus AMPH; ^δ^PEGyAMPH versus AMPH. Data presented as mean ± SEM. See also Figure S5.

Recent evidence from our group and others (Camell et al., 2017, Pereira et al., 2017, Pirzgalska et al., 2017) demonstrates that peripheral NE regulates adiposity levels independent of FI or exercise. As such, we hypothesized that PEGyAMPH treatment could promote long-term anti-obesity results, regardless of excessive caloric intake. To investigate this, we exposed adult C57BL/6 male mice to HFD and treatment with either AMPH or PEGyAMPH (120 μmol/kg of BW for both drugs or control PBS, daily i.p. injections). As demonstrated before, AMPH protects mice from DIO (Figures 5A and 5B), while PEGyAMPH’s *sympathofacilitator* activity is indeed sufficient to protect BW in a dose-dependent manner (Figure S3G). Notably, when administrated in equimolar dose, treatment with PEGyAMPH showed similar size effect on promoting leanness under HFD exposure to that of its unmodified counterpart (Figures 5A and 5B).

Moreover, both therapies also improved peripheral insulin sensitivity, as blood glucose levels did not differ between all groups (Figure S5A), but insulin was significantly lower in the treated groups compared with those of the PBS controls (Figure S5B). Given that the SNS is reported not only to control insulin sensitivity but also its secretion (Morton et al., 2017, Nonogaki, 2000, Ruud et al., 2017), we quantified NE content in the pancreas and observed that only AMPH increased SNS tone in this tissue (Figure S5C). Thus, PEGyAMPH treatment prevents the development of hyperinsulinemia and improves glucose homeostasis by increasing peripheral insulin sensitivity without suppressing secretion. Analysis of liver gene expression revealed that both treated groups had a 2-fold elevation of phosphoenolpyruvate carboxykinase (*PEPCK*) expression (Figure S5D), which is a main integrator of energy metabolism (Burgess et al., 2007, Petersen et al., 2017, She et al., 2000). Of note, the higher insulin sensitivity found in treated animals during the fed-state was not associated with major differences in glucose levels during an i.p. glucose tolerance test (GTT) (Figures S5E and S5G). Yet, the PEGyAMPH-treated group revealed a trend toward higher peripheral glucose uptake during an insulin tolerance test (ITT) (Figures S5F and S5H). Combined, these results indicate that long-term treatment with PEGyAMPH protects mice from DIO and improves glucose homeostasis during HFD exposure.

As expected, PEGyAMPH-treated mice did not decrease their FI (Figure 5C) or increase LA (Figures 5D and S5H) upon chronic treatment. Nonetheless, indirect calorimetry revealed that, under HFD feeding, this group had significantly higher energy expenditure (EE) compared with the PBS controls (Figures 5E and S5G), despite having similar behavior. We then analyzed the effects of PEGyAMPH treatment on dietary lipid absorption and found that it did not alter the total 24-h fecal output or its lipid content (Figure 5F). Plasma TGs levels of PEGyAMPH-treated mice were also unchanged relative to controls (Figure 5G); thus, we can conclude that PEGyAMPH promotes leanness by overriding caloric intake. Finally, to test the efficacy of the treatments for weight loss of already-obese animals, we also treated C57BL/6 mice previously exposed for minimum of 3 months to HFD, with either AMPH or PEGyAMPH (120 μmol/kg of BW, i.p.) and found that both drugs caused significant weight loss (>10%) after just 3 weeks of daily injections (Figures 5H and 5I).

Altogether, our results indicate that treatment with PEGyAMPH during HFD exposure overrides FI by increasing EE and adrenergic-stimulated metabolic pathways.

### PEGyAMPH Protects from Obesity by Elevating Lipolysis and Lipid Utilization

**Figure 6:**
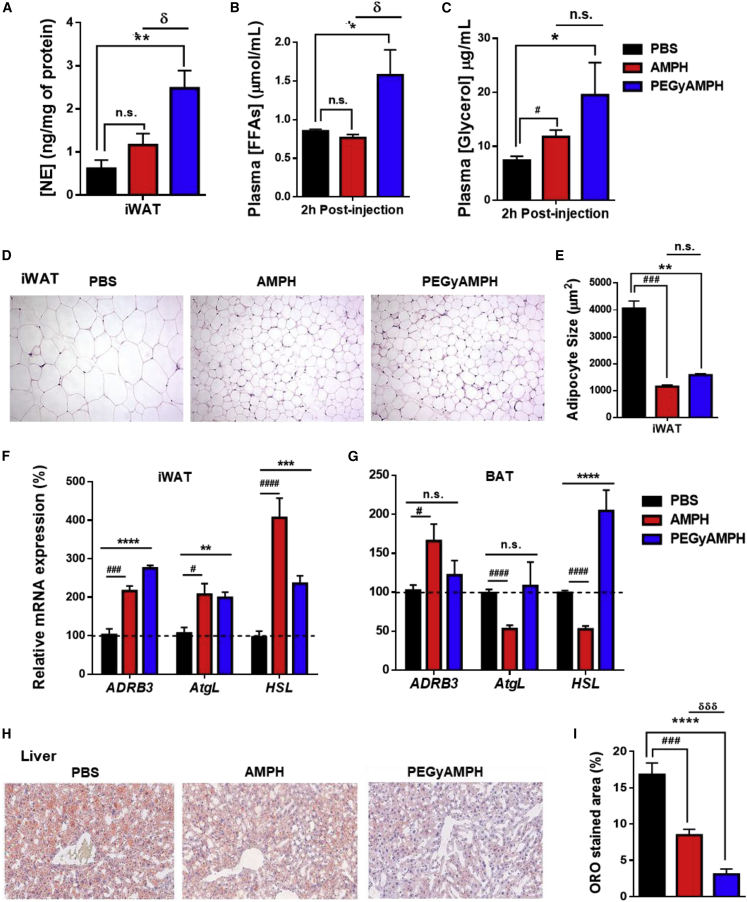
PEGyAMPH Elevates Adipose Tissue Lipolysis and Peripheral Lipid Utilization during DIO (A–C) NE content in iWAT after 10 weeks of HFD exposure and respective treatment (A). Plasma levels of FFAs (B) and glycerol (C). (D and E) Representative histology of iWAT stained with H&E (D) and quantification of iWAT adipocyte size (E). (F and G) Lipolytic gene expression in iWAT (F) and in BAT (G) determined by qRT-PCR relative to housekeeping gene *Arbp0*. (H) Representative histology of liver oro staining. (I) Quantification of oro staining normalized to the total liver area. (n = 5–12; statistics done using unpaired Student’s t test, with Holm-Sidak correction). ^∗,#,δ^p < 0.05; ^∗^PBS versus PEGyAMPH; ^#^PBS versus AMPH; ^δ^PEGyAMPH versus AMPH. Data presented as mean ± SEM. See also Figure S6.

As mentioned above, we found that PEGyAMPH’s anti-obesity action is dose dependent as are its *sympathofacillitor* properties (Figures 3E and S3G, respectively). Thus, to dissect its metabolic effects, we started by analyzing adipose SNS tone after 10 weeks of HFD exposure and treatment. Surprisingly, the PEGyAMPH-treated group exhibited a much greater NE content in iWAT compared not only to the control group but also to that of AMPH-treated animals (Figure 6A). This was also associated with the presence of higher levels of lipolytic markers in circulation, namely, free fatty acids (FFA) and glycerol (Figures 6B and 6C), highlighting the potential of PEGyAMPH for chronic treatment. We observed a marked reduction in iWAT adipocyte size (Figures 6D and 6E) in both treated groups relative to the same depot in PBS-treated animals. Next, we probed changes of gene expression in several metabolic tissues. PEGyAMPH induced an almost 3-fold upregulation in β_3_-adrenoceptor (*ADRB3*) expression in iWAT, but not in BAT (Figures 6F and 6G). This was accompanied by a doubling of adipose triglyceride lipase (*AtgL*), while hormone-sensitive lipase (*HSL*) showed increased expression in both iWAT and BAT (Figures 6F and 6G). Combined with the upregulation of adipose lipolysis, SNS tone was also found to be elevated in liver and skeletal muscle (Figures S6A and S6D, respectively) after treatment with PEGyAMPH, which suggests higher metabolic performance, i.e., higher utilization of lipid stores (Geerling et al., 2014, Nonogaki, 2000). In line with this, we found decreased TG content accompanied by an increase of glycogen (Figures S6B and S6C) in the livers of PEGyAMPH-treated mice. By performing oil red O (ORO) staining and quantification (Figures 6H and 6I), we confirmed a marked reduction in hepatic steatosis. In the skeletal muscle, PEGyAMPH caused a similar reduction of TG levels while preserving glycogen stores (Figures S6E and S6F). Quantification of gene expression in both liver and muscle confirmed alterations of lipid metabolism in these tissues (Figures S6G and S6H).

Hence, PEGyAMPH’s protection against DIO seems to be associated with a general elevation of peripheral lipid breakdown and utilization, highlighting the SNS as a major regulator of adiposity during excessive caloric intake.

### PEGyAMPH Protects from Obesity by Elevating Thermogenesis and Heat Dissipation via ADRB2

**Figure 7:**
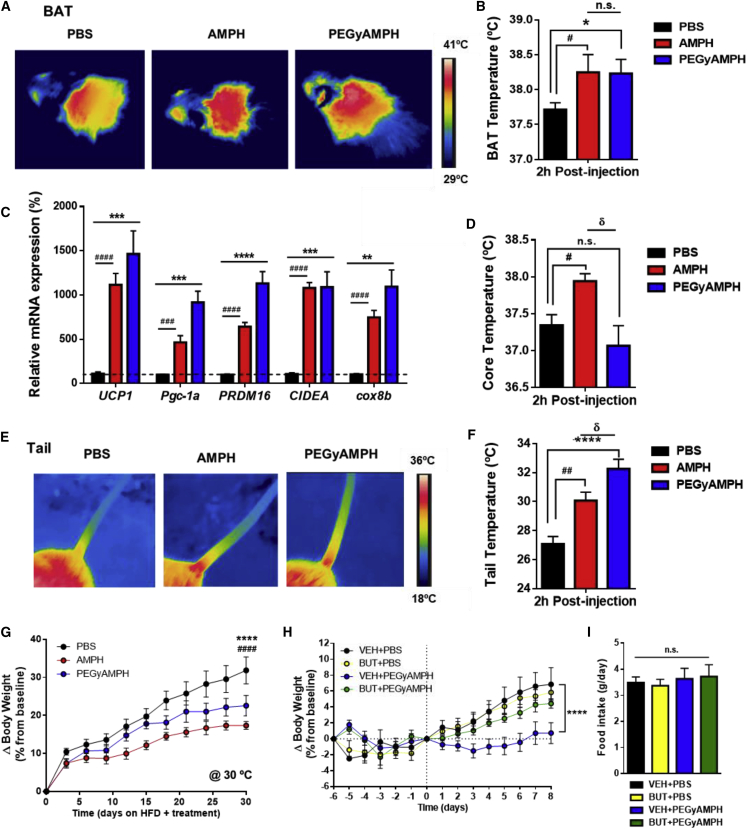
PEGyAMPH Increases Thermogenesis and Heat Dissipation, and it Protects against Obesity via ADRB2 (A) Representative infrared thermography of the BAT area. (B) Quantification of BAT skin temperature post-injection with PBS, AMPH, or PEGyAMPH (120 μmol/kg of BW, i.p.). (C) BAT mRNA levels of thermogenic genes determined by qRT-PCR relative to housekeeping gene *Arbp0*, after 10 weeks of HFD exposure and treatment. (D) Core body temperature measured with rectal probe. (E) Representative infrared thermography of tail. (F) Quantification of tail temperature measured 0.5 cm from the base. (n = 8–12; statistics done using unpaired Student’s t test, with Holm-Sidak correction). (G) ΔBW of mice exposed to HFD and treatment with PBS, AMPH, or PEGyAMPH under thermoneutral housing conditions. (H and I) ΔBW (H) and daily FI of mice exposed to HFD and treatment with PBS or PEGyAMPH in combination with BUT (16 μmol/kg/day, delivered via osmotic pumps) (I). (n = 10–12; statistics done using two-way ANOVA). ^∗,#,δ^p < 0.05; ^∗^PBS versus PEGyAMPH; ^#^PBS versus AMPH; ^δ^PEGyAMPH versus AMPH. Data presented as mean ± SEM. See also Figure S7.

Activation of thermogenesis is also controlled by the SNS and can act as an energy *sink*, promoting resistance to obesity (Rothwell and Stock, 1979). Nonetheless, the rapid increase in EE observed upon AMPH administration could be a result of increased LA, and thus the contribution of its thermogenic activity to the elevation of metabolic rate and BW management is still debated (Arch and Trayhurn, 2013). To assess the thermogenic effect of each drug, we used infrared thermography to analyze BAT temperature. After PEGyAMPH treatment, there was an elevation in BAT temperature similar to that evoked by AMPH (Figures 7A and 7B). Accordingly, we also found that both amphetamines caused a 15-fold upregulation of the primary BAT thermogenic marker, uncoupling protein 1 (*UCP1*), and increased all other thermogenic genes probed (Figure 7C). This was accompanied by an 8-fold increase in the expression of glucose-transporter-type-4 isoform (*GLUT4*) (Figure S7A), which indicates higher glucose uptake by this organ being a marker of higher thermogenic activity (Lee et al., 2016), possibly accounting for the increased insulin sensitivity compared with the PBS-treated controls (Figure S5B). Although *UCP1* levels were not changed in iWAT, all other thermogenic genes quantified were upregulated (Figure S7B), and others have reported thermogenesis with invariant UCP1 (Granneman et al., 2003, Ikeda et al., 2017, Ikeda et al., 2018). The combination of these results reveals a general trend for elevated thermogenesis after PEGyAMPH treatment, which underlies its protection against DIO.

Surprisingly, we also detected that only AMPH caused transient hyperthermia (Borbély et al., 1974), while PEGyAMPH-treated mice remain normothermic (Figure 7D). This suggests that, although both drugs act as *sympathomimetics*, they might actually have different actions on peripheral vasculature and heat dissipation (Blessing et al., 2016). To assess vasoconstriction at the extremities, we probed the local temperature at the tail base by thermography (Fischer et al., 2016, Warner et al., 2013), and found that, despite the similar core temperature, PEGyAMPH-injected mice had significantly warmer tails relative to those of the controls (Figures 7E and 7F). This indicates that, unlike AMPH, PEGyAMPH’s *sympathomimetic* activity increases thermogenesis without causing vasoconstriction, emphasizing thermoregulation as a relevant component of adiposity control (Jéquier et al., 1974, Kasza et al., 2019, Warner et al., 2013). Given that PEGyAMPH-treated mice rely on activation of thermogenesis to remain lean, we tested the effect of the drug at thermoneutrality. As expected, PEGyAMPH anti-obesity potency is decreased in this environmental setting, yet some level of protection against DIO does remain (Figures 7G and S7E). Furthermore, while thermogenesis is shown to be important but not necessary for PEGyAMPH’s action, its facilitation of heat dissipation seems to be driven by vasodilation, a process well known to be controlled by ADRB2 (Chruscinski et al., 1999, Ernande et al., 2016). Consistently, we discovered that the anti-obesity effect of PEGyAMPH is abrogated by selective ADRB2 antagonism (BUT, Figures 7H and 7I), further validating the importance of this pathway for PEGyAMPH’s metabolic effects.

Altogether, these results confirm that PEGyAMPH is a peripheral *sympathofacilitator* anti-obesity drug that activates a whole-body energy *sink* by coupling thermogenesis to heat dissipation, without inducing behavioral changes or cardiotoxicity.

## Discussion

The primary mechanism of action that underlies the anti-obesity effect of AMPH-based drugs, such as FDA-approved phentermine, is based on an effect in the brain that conveys pronounced behavioral effects: anorexia and hyperkinesia. Phentermine is a centrally acting anorexigenic drug that was developed as a less addictive option to other AMPH forms. However, studies in rodents have suggested that the anti-obesity effects of AMPHs and other anorexigenic drugs are partly, or even entirely, a result of non-behavioral factors (Arch, 1981, Herling et al., 2008). Although anorexia unquestionably reduces BW, our results indicate that this effect depends on an intact sympathetic axis (Pereira et al., 2017). And as ADRB3 was described to be the main receptor mediating adrenergic-stimulated lipolysis in rodent adipocytes (Bloom et al., 1992, Guerra et al., 1998, Himms-Hagen et al., 1994, Susulic et al., 1995, Xiao et al., 2015), direct sympathomimetic agents, such as the ADRB3 agonist CL-316,243, were once regarded as potential anti-obesity therapies. However, as human lipolysis is mainly mediated by the other β-adrenoreceptors, ADRB3 agonists failed as anti-obesity therapies (Arch, 2011, Lafontan and Berlan, 1993, Ursino et al., 2009). Direct thermogenic drugs, such as compound 2,4-dinitrophenol, a mitochondrial uncoupler, were very effective anti-obesity treatments through converting energy to heat, but they also cause substantial side effects, including life-threatening hyperthermia (Harper et al., 2001). The historical failure of post-synaptic targeting in adipose tissue is suggestive of an orchestrated multi-pathway and multi-organ program that is pre-synaptically controlled by the SNS (Bartness et al., 2014, Mahú and Domingos, 2017, Zeng et al., 2015). Thus, we reasoned that a pre-synaptic facilitation of sympathetic output would have a more potent effect, as SNS circuits would simultaneously activate multiple pathways, not only in WAT, but also in BAT and other metabolically relevant organs. Indirect sympathomimetics, such as phentermine, demonstrated higher anti-obesity efficacy relative to the direct class but have prohibitive cardio-excitatory effects. However, whether this side effect is mediated via the brain or periphery had never been experimentally tested, and we addressed that here by performing i.c.v. injections of AMPH and confirming the central control of the cardiovascular effects. As such, we reasoned and demonstrated that an exclusively peripheral *sympathofacilitator*, PEGyAMPH, which does not enter the brain, could be devoid of cardiovascular side effects. PEGyAMPH reduces obesity with a size effect comparable to that of AMPH, yet with a distinct mechanism in that it spares effects relating to brain action, such as anorexia, hyperkinesia, and tachycardia.

Engagement of ADRB2 seems to be required for the *sympathofacilitatory* and weight-loss effects of this drug. And elevation of SNS tone, both to WAT and BAT, activates lipolysis and thermogenesis (Bartness et al., 2014, Contreras et al., 2017, Hausberg et al., 2002, Mahú and Domingos, 2017, Zeng et al., 2015). Simultaneously, ADRB2 agonism is well known to lead to smooth muscle relaxation and vasodilation, which bode well to mediate the cardioprotective actions of PEGyAMPH, as well as its effect on thermal dissipation. Consistently, others have shown that the ADRB2-selective agonist salbutamol, increases BAT vasodilation and tissue perfusion, activating thermogenesis without directly targeting brown adipocytes (Ernande et al., 2016). The authors of this report did not assess changes in peripheral vasculature, and we did not probe BAT perfusion, but it is quite possible that PEGyAMPH might also increase blood flow to this tissue, further boosting thermogenesis. Moreover, although the relationship between peripheral vascular tone and cardiac function is undebatable (Delong and Sharma, 2019), the effect of blood flow on heat dissipation and its connection to metabolic regulation has only recently began to be appreciated as an important component of adiposity control. In fact, compensatory thermoregulation seems to drive hypermetabolic phenotypes in animals with genetic manipulations that facilitate heat dissipation (Kasza et al., 2019, Reimúndez et al., 2018, Warner et al., 2013).

Our results put forward the idea that coupling increases thermogenesis with peripheral heat dissipation, constitutes a *sink* for EE without causing hyperthermia, and that AMPH-like compounds, such as phentermine or ADRB2 agonists, which are not indicated for long-term systemic use due to serious side effects, could be reformulated to become brain impermeable. Overall, our results are a proof-of-principle that peripheral *sympathofacilitators* could be a new generation of anti-obesity compounds that circumvent difficulties caused by BBB permeability and avoid brain-related side effects, including those relating to cardiovascular function.

### Limitations of Study

Considering the underappreciated promiscuity of several FDA-approved drugs, which can bind, on average, to 12 or more distinct targets (Reker et al., 2014a, Reker et al., 2014b, Rodrigues et al., 2016), we cannot exclude that PEGyAMPH, or even AMPH, binds a plethora of targets not covered in this study. Notwithstanding this limitation, PEGyAMPH’s effect on weight requires a functional ADRB2, and its cardiac side effects require access to the brain. Systemically administered ADRB2 agonists have been illicitly used for weight management and their cardiac side effects have long been a public health and safety concern (Salpeter, 2004, Spiller et al., 2013). Whether brain-sparing agonists phenocopy the anti-obesity and cardioprotective effects of PEGyAMPH will have to be experimentally tested—the loss of function of ADRB2 in this study is limited to demonstrating necessity and does not predict the outcome of a gain of function of ADRB2 based on peripherally restricted agonists.

## STAR★Methods

### Key Resources Table

**Table:** 

REAGENT or RESOURCE	SOURCE	IDENTIFIER
**Antibodies**		

Rb Anti-Tyrosine Hydroxylase Antibody	Milipore	Cat#AB152; RRID:AB_390204
Alexa Fluor® 594 Goat Anti-Rabbit IgG (H+L) Antibody	Life Technologies	Cat#A-11012; RRID:AB_2534079

**Chemicals, Peptides, and Recombinant Proteins**		

Diphtheria Toxin, Unnicked, *Corynebacterium diphtheriae*	Sigma-Aldrich	Cat#322326
*l*-Amphetamine - (2R)-1-phenylprop-2-ylamine HCl salt	Asiba Pharmatec, Inc	Cat#10296-HCl
PEGylated *l*-Amphetamine	Wuxi AppTec	Cat#IGC-20180911
Butoxamine hydrochloride	Santa Cruz Biotechnology	Cat#sc-234233
MS(PEG)4 Methyl-PEG-NHS-Ester	Life Technologies	Cat#22341
Acetylcholine chloride	Sigma-Aldrich	Cat#A6625
Collagenase from *Clostridium histolyticum*	Sigma-Aldrich	Cat#C2674
D-glucose	Sigma-Aldrich	Cat#G-8270
0.25% trypsin solution	Biowest	Cat#L0931
Poly-D-Lysine solution	Sigma-Aldrich	Cat#A-003-M
BD Matrigel Matrix	BD Biosciences	Cat#356234
Neurobasal Medium	Gibco	Cat#10888022
B-27	Gibco	Cat#17504044
Nerve Growth Factor	AbD Serotec	Cat#NC0419584
5-Fluoro-2′-deoxyuridine	Sigma-Aldrich	Cat#856657
*N*,*N*-Diisopropylethylamine (DIPEA)	Sigma-Aldrich	Cat#387649
Insulin	Sigma-Aldrich	Cat#91077C

**Critical Commercial Assays**		

Noradrenaline Research ELISA	LDN Labor Diagnostika Nord GmbH & Co.KG	Cat#BAE-5200
Triglyceride Quantification Kit	Abcam	Cat#AB65336
Free Fatty Acid Quantitation Kit	Sigma-Aldrich	Cat#MAK044
Free Glycerol Reagent	Sigma-Aldrich	Cat#F6428
Glycerol Standard Solution	Sigma-Aldrich	Cat#G7793
Glycogen Assay Kit	Abcam	Cat#AB65620
RNeasy Plus Micro Kit	Qiagen	Cat#50974034
PureLink RNA Mini Kit	Life Technologies	Cat#12183025
Power SYBR Green PCR Master Mix	Invitrogen	Cat#4368706
SuperScript II Reverse Transcriptase	Invitrogen	Cat#18064-014
RNaseOUT Recombinant Ribonuclease Inhibitor	Invitrogen	Cat#10777-019
Mouse Ultrasensitive Insulin ELISA	Alpco	Cat#80-INSMSU-E01
Protein Assay Dye Reagent Concentrate	Bio-Rad	Cat#5000006

**Deposited Data**		

Coordinates of the Minimized Complexes and input files	Dropbox	https://bit.ly/2TJvCgA

**Experimental Models: Organisms/Strains**		

Mouse: C57BL/6. C57BL/6J	Charles River	jax.org/strain/000664
Mouse: TH-cre. B6.Cg-*7630403G23Rik*^*Tg(Th-cre)*1*Tmd*^/J	The Jackson Laboratory	Cat#008601
Mouse: CAG-LSL-*GCaMP3*. B6;129S-*Gt*(ROSA)*26Sor*^*tm38(CAG-GCaMP3)Hze*^/J	The Jackson Laboratory	Cat#014538
Mouse: LSL-DTR. C57BL/6-*Gt(ROSA)26Sor*^*tm1(HBEGF)Awai*^/J	The Jackson Laboratory	Cat#007900
Mouse: GCaMP3^+^ B6;*TH-cre*; CAG-LSL*-GCaMP3*	This paper	N/A
Mouse: B6;*TH-cre*; LSL-DTR	Pereira et al., 2017	N/A

**Oligonucleotides**		

N/A	Table S1 for qPCR primers sequences	N/A

**Software and Algorithms**		

AMBER18 -Molecular Mechanics Minimizations	AMBER18	http://ambermd.org
Prism	GraphPad Software	https://www.graphpad.com/scientificsoftware/prism/
CODA Software	Kent Scientific Corporation	https://www.kentscientific.com/products/coda-data-acquisition-software/
MouseOx Plus Software	Starr Life Sciences Corp.	https://www.starrlifesciences.com/resource/pulse-oximetry-software-update/
Avidemux Software - Video processing	Avidemux.Org	Avidemux 2.7.1
TrackMate	Fiji	https://imagej.net/TrackMate
FLIR-Tools Software	FLIR	https://www.flir.com/products/flir-tools/
MetaFluor Fluorescence Ratio Imaging Software	Molecular Devices	N/A
Docking calculations	PatchDock	https://bioinfo3d.cs.tau.ac.il/PatchDock/php.php
Docking calculations - refinement results	FireDock	http://bioinfo3d.cs.tau.ac.il/FireDock/php.php

**Other**		

InfraRed Camera	FLIR	Cat#E75
Noninvasive Blood Pressure System - Volume Pressure Recording (VPR)	Kent Scientific Corporation	CODA High Throughput System
Thermometer with rectal probe	Physitemp	Cat#BAT-12; Cat#RET-3
ACCU-CHEK Aviva Glucose meter and strips	Roche Sistemas de Diagnósticos Lda	Cat#06453970023
High Fat Diet	Ssniff, Spezialdiäten	Cat#D12492
MouseOx Plus System with CollarClip Sensor	Starr Life Sciences Corp.	N/A
Calorimetric System LabMaster	TSE Systems	N/A
Toshiba Camileo HD	Toshiba	Cat#PA4083E-1CAM
ABI QuantStudio 7	Applied Biosystems	Cat#4485701
Axiovert 135 TV	Zeiss	N/A
Toohey Spritzer pressure system IIe fluid delivery system	Toohey Company	N/A
CoolSNAPfx CCD Camera	Photometrics	N/A
Axopatch 200B Microelectrode Amplifier	Axon Instruments	N/A
FTICR mass spectrometer - *Bruker Apex Ultra* with a 7 Tesla actively shielded magnet	Bruker	N/A
Eppendorf Concentrator Plus system	Eppendorf	Cat#5305000100
Shimadzu HPLC System	Shimadzu UK Limited	N/A
ACQUITY UPLC BEH C18 Column	Waters	Cat#186002350
Exactive Orbitrap Mass Spectrometer	Thermo Fisher Scientific	Cat#IQLAAEGAAPFALGMBCA
Micro-Osmotic PUMPs	Alzet	Cat#1004
Temperature controlled Incubators	Aralab	Cat#FITOCLIMAS600PLHV

### Resource Availability

#### Lead Contact

Further information and requests for resources should be directed to and will be fulfilled by the Lead Contact, Ana I. Domingos (ana.domingos@dpag.ox.ac.uk). All data that support the findings herein presented are available from the corresponding authors upon reasonable request.

#### Materials Availability

We developed a PEGylated version of amphetamine, for which the reaction protocol is described in the methods section. The modified drug can be produced and purchased at WuXi AppTech upon request for research purposes only.

#### Data and Code Availability

The results generated using PatchDock web server are available at: [http://bioinfo3d.cs.tau.ac.il/PatchDock/runs/6H7J_receptorH.pdb_PEGyAMPH.pdb_19_56_7_10_2_120/; http://bioinfo3d.cs.tau.ac.il/PatchDock/runs/6H7J_receptorH.pdb_AMPH.pdb_50_0_8_10_2_120/]. The results generated using FireDock web server are available at: [http://bioinfo3d.cs.tau.ac.il/FireDock/runs/6H7J_receptorH.pdb_PEGyAMPH.pdb_28_57_7_10_2_120/; http://bioinfo3d.cs.tau.ac.il/FireDock/runs/6H7J_receptorH.pdb_AMPH.pdb_27_1_8_10_2_120/]. Original data related to the coordinates of the Minimized Complexes and input files have been deposited to Mendeley Data [https://doi.org/10.17632/cxx6gy2rjx.2] and are accessible in the dropbox link found in the Key Resources Table.

### Experimental Model and Subject Details

#### Mice and Housing Conditions

Mice were housed at controlled temperature and humidity, under a 12 h light/dark cycle. Food and water were supplied *ad libitum*, unless mentioned otherwise. All animal protocols were approved by the Instituto Gulbenkian de Ciência ethical committee and the “*Órgão Responsável pelo Bem-estar dos Animais*” (ORBEA). These were consequently licensed by the Direccão Geral de Alimentação e Veterinária (DGAV - Project ID 15010/14/006). All experimental procedures follow the Portuguese (Portaria n° 1005/92, Decreto-Lei n° 113/2013) and European (Directive 2010/63/EU) legislations, concerning housing, husbandry and animal welfare. C57BL/6J mice were obtained from Charles River and bread in the Mice Production Facility at the IGC. *TH-cre* (Jax, #008601), CAG-LSL-*GCaMP3* (Jax, #014538), LSL-DTR (Jax, #007900), mice were purchased from Jackson Laboratory, and bred inhouse to produce homozygous *TH-cre;* CAG-LSL*-GCaMP3* and *TH-cre;* LSL-DTR mice. LSL-DTR mice were used as controls for the sympathectomization studies.

### PEGyDT-Mediated Regional Sympathectomy

For detailed characterization refer to Pereira et al., 2017. Briefly, reconstitute Diphtheria Toxin (DT - Sigma) and MS(PEG)4 Methyl-PEG-NHS-Ester (Life Technologies) according to manufacturer instructions. For each 1 g of DT add 0.423 mg of PEG and incubate the reaction for 4 h at RT under agitation using a shaker to produce the stock solution of PEGylated DT (PEGyDT). 7-8 weeks old *TH-cre;* LSL-DTR male mice were used for genetic-sympathectomy experiments and aged-matched LSL-DTR were used as controls. PEGyDT was administered once a day for 8 consecutive days (25 ng/g of BW, i.p. injections, diluted in PBS). All following experiments were performed at least 24 h after the last injection.

#### High-Fat Diet Challenge and Chronic Treatments

All mice used for DIO challenges and follow-up metabolic analysis were males. When C57BL/6 male mice reached 8 weeks of age, or 1 day after sympathectomy protocol was performed in both *TH-cre; LSL-DTR* and respective controls *LSL-DTR*, normal diet was replaced with high fat diet (Ssniff Spezialdiäten, D12492) concomitantly with treatment of either AMPH or PEGyAMPH (dose: 120 μmol/kg of BW for both drugs diluted in PBS, daily i.p. injections). Length of exposure to HFD and treatment is indicated in figure legends. Butoxamine was delivered via micro-osmotic pumps (dose:16 μmol/kg/day), implanted intraperitoneally 1 week before treatment with PEGyAMPH (Alzet).

#### SCG Neurons Culture and Treatments

Primary cultures of SCG neurons were performed from postnatal day 30 C57BL/6 or *TH-cre;* CAG-LSL*-GCaMP3* mice (male and female). After decapitation, both SCG of each animal were removed and cleaned of all visible adipose tissue and surrounding connective tissue before transfer to Dulbecco's Modified Eagle Medium (Biowest). Then, SCG were treated enzymatically in two steps to yield single neurons in accordance to the method described by Motagally and collaborators (Motagally et al., 2009), with some modifications. First, SCG were subjected to enzymatic dissociation in 2.5 mg/mL collagenase solution (Sigma-Aldrich) in Hank's Balanced Salt Solution (HBSS) without calcium and magnesium (Gibco, Life Technologies) at 37°C with agitation, followed by 0.25% trypsin solution (Biowest) in PBS at 37°C with agitation. SCG was then mechanically dissociated into a suspension of single cells. The isolated sympathetic neurons were plated, 2500 cells per coverslip (6 mm) coated with poly-d-lysine (Sigma) and growth factor-reduced Matrigel (BD Biosciences) and cultured in Neurobasal medium (Gibco) supplemented with 2% B-27 (Gibco), 10% fetal bovine serum (Gibco), 1% penicillin/streptomycin (Biowest), 100 ng/mL nerve growth factor (AbD Serotec) and 5 μM 5-fluoro-2'-deoxyuridine (Sigma-Aldrich). Cells were kept in culture for 6 days *in vitro* at 37°C with 5% CO_2_ conditioned atmosphere to obtain an enriched culture of sympathetic neurons. Before measurements, neurons were incubated with 15 μM AMPH or 15 μM PEGyAMPH for 24 h at 37°C with 5% CO_2_ conditioned atmosphere. Butoxamine (Santa-Cruz Biotechnology) was add at a concentration of 10μM, 30min prior to the calcium-imaging experiments.

### Method Details

#### PEGylation of Amphetamine (PEGyAMPH Synthesis)

Inspired by Yang et al. (2009). Briefly, in a round-bottom flask *(R)-*1-phenylprop-2-ylamine hydrochloride salt (103 mg, 0.6 mmol, 2 equiv., Asiba Pharmatec.) was placed under inert atmosphere. A solution of methyl-PEG-NHS-ester reagent (1.1 mL, 100 mg, 0.39 mmol, 1 equiv., Thermo Scientific) in DMSO was then added, followed by the addition of diisopropylethylamine (DIPEA, 105 μL, 0.6 mmol, 2 eq, Sigma-Aldrich). The reaction was stirred at room temperature for 46 h, after which a multiple extraction with water/ethyl acetate was performed to remove the product from DMSO. Then preparative chromatography (5% EtOAc in MeOH, v/v) was performed to isolate compound PEGyAMPH in 98% yield (0.1 g). Characterization: ^**1**^**H NMR (300 MHz, CDCl**_**3**_**)** δ 7.25 – 7.11 (m, 5H), 6.53 – 6.26 (m, 1H), 4.19 (p, *J* = 6.8 Hz, 1H), 3.63 – 3.47 (m, 14H), 3.32 (s, 3H), 2.79 (dd, *J* = 13.5, 6.1 Hz, 1H), 2.65 (dd, *J* = 13.5, 7.1 Hz, 1H), 2.37 (t, *J* = 6.4 Hz, 2H), 1.06 (d, *J* = 6.6 Hz, 3H) ppm. ^**13**^**C NMR (75 MHz, CDCl**_**3**_**)** δ 170.92, 138.38, 129.55, 128.36, 126.40, 72.01, 70.70, 70.60, 70.46, 70.34, 67.43, 59.11, 46.02, 42.60, 37.21 ppm. **HRMS**: [M+H]^+^_*calc*_ = 354.22750; [M+H]^+^_*real*_ = 354.22783 (error –0.9 ppm). Scale-up of the reaction for the chronic *in vivo* treatments was reproduced by Wuxi AppTec.

#### Intracerebroventricular Treatments

Intracerebroventricular (i.c.v.) cannulae were stereotaxically implanted under a mix of inhaled isoflurane and oxygen, using the following coordinates 1.5 mm lateral to bregma, 0.6 mm posterior, 4.0 mm deep. Mice equipped with i.c.v. cannulae were given 7 days to recover before injections and measurements. A bolus i.c.v. injection of AMPH or PEGyAMPH (60 nmol, diluted in 5 μL of PBS), or of PBS as control was acutely administrated for behavioral and cardiorespiratory measurements as described below.

#### Non-invasive Cardiovascular Measurements

Blood Pressure and Heart Rate were measured from awake restrained animals using a Volume Pressure Recording (VPR) sensor and tail-cuff system (CODA, Kent Scientific Corporation). To prevent stress-related effects, mice were trained for a minimum of 3 days before measurements. At least 15 accurate measurements per animal were used for analysis of diastolic, mean and systolic pressure and at least 8 for analysis of the heart rate. Baseline was recorded just before injection, and the effect of the drugs was measured 15-30 min post injection with PBS, AMPH or PEGyAMPH (dose: 120 μmol/kg of BW for both drugs, daily i.p. injections).

#### Infrared Pulse Oximetry

The day before measurements the hair around the neck of each mouse was removed using Veet cream (Unilever). 24-48h post-depilation, the cardiopulmonary status of each mouse was analyzed by MouseOx Plus (Starr Life Sciences Corp) in accordance with manufacturer's instructions. Each mouse was very briefly anaesthetised using 5% isoflurane to facilitate placement of a CollarClip Sensor, and allowed to acclimatize to the anesthesia with 1-2% isoflurane for 5 min. This time window was sufficient for animals to recover normal activities and physiological readings. Measurements were then recorded for 5-10 min at baseline and then for another 10-15min after injections (i.p. of PEGyAMPH and AMPH, dose: 120 μmol/kg of BW for both drugs; or i.c.v. of PBS and AMPH, dose: 60nmol, bolus per animal). The time points described were used to collect representative, error-free data due to the motion artefact (DeMeulenaere, 2007).

#### Locomotion Assays

After 3 weeks of HFD exposure and treatment, mice were acclimated to tracking cages for 1 week before starting the 72h locomotion measurements by using a high throughput tracking system (LabMaster, TSE Systems). Animals were also filmed for 20-30 min, with a Toshiba Camileo HD camera 1 h post-injection inside their normal housing cage, for assessment of total distance travelled. Footage-records were filtered by using video editor Avidemux (Avidemux 2.7.1) and 10-15 min distance computations were quantified with the TrackMate tracking plugin from Fiji (Fiji; Wisconsin-Madinson).

#### Calorimetry Assays

Animals were analysed for Energy Expenditure (EE) using a calorimetric system (LabMaster; TSE Systems). Animals were placed in a temperature-controlled (24°C) box through which air was pumped. After calibrating the system with the reference gases (20.9% O_2_, 0.05% CO_2_ and 79.05% N_2_), the metabolic rate was measured for 2-3 days, and EE was recorded every 30 min. Animals were placed for adaptation for 1 week before starting the measurements. Normalized EE was calculated as described in Tschöp et al. (2011) and the distribution curves were obtained using the CalR Web-based tool (Mina et al., 2018).

#### Glucose Metabolism Tests

For the intraperitoneal Glucose Tolerance Test (GTT), mice were injected with PBS, AMPH or PEGyAMPH and then fasted for 6 h, before being given 2 g glucose/kg of BW, i.p. Blood was drawn from the tail vein and glucose levels were measured using a glucometer (Accu-Check System, Roche) at 0, 15, 30, 60, 90, and 120 min after glucose administration. For the Insulin Tolerance Test (ITT), mice were injected with PBS, AMPH or PEGyAMPH and then fasted for 2 h, before being given (i.p.) 0.9U/kg of BW, i.p., of recombinant human insulin (Sigma), blood glucose levels were measured at 0, 15, 30, 60, 90, 120, 150 and 180 min after insulin administration.

#### Thermoregulation Analysis

All measurements were done in *ad libitum* fed mice 2 h post-injections with PBS, AMPH or PEGyAMPH. Rectal temperature was measured with an electronic thermometer (Physitemp). BAT and Tail thermographic pictures were taken with a Compact-Infrared-Thermal-Imaging-Camera (FLIR) and FLIR-Tools-Software (FLIR), to quantify local temperature (Martínez-Sánchez et al., 2017).

#### Blood and Plasma Analysis

Blood was collected from the tail vein of HFD fed mice 2 h post-injections with PBS, AMPH or PEGyAMPH, without access to food. Blood glucose was measured with a glucometer (Accu-Check, Roche). Analysis of Insulin, Triglycerides, Glycerol and FFA levels in plasma was performed by using Mouse Ultrasensitive Insulin ELISA (Alpco), Triglyceride Quantification Kit (Abcam), Free Glycerol Reagent (Sigma) and Glycerol Standard Solution (Sigma), and Free Fatty Acid Quantification Kit (Sigma), respectively according to manufacturer’s instructions.

#### Tissue NE Measurements (ELISA)

To assess peripheral NE content in tissues, mice were sacrificed in *ad libitum* conditions 2 h post injection with PBS, AMPH or PEGyAMPH. NE levels were determined with a NE ELISA kit (Labor Diagnostika Nord GmbH). Tissues were homogenized and sonicated in homogenization buffer (1 M HCl, 1 mM EDTA, 4 mM sodium metabisulfite), and cellular debris was pelleted by centrifugation at 20,000 g for 10 min at 4°C). All tissue samples were normalized to total tissue protein concentration, measured with Protein Assay Dye Reagent Concentrate (Bio-Rad), according to manufacturer’s instructions.

#### Fecal Output Assay

24 h fecal output was collected and weighed. The feces were washed with 1x PBS and total triglyceride content was extracted by homogenization and boiling, for 2 cycles of 5 min, in 5% NP-40. Triglyceride content was measured using Triglyceride Quantification Kit (Abcam), according to manufacturer’s instructions, and normalized to the weight of total fecal output.

#### Tissue Triglycerides Analysis

To assess gastrocnemius muscle and liver content in tissues, mice were sacrificed in *ad libitum* conditions 2 h post injection with PBS, AMPH or PEGyAMPH. Triglyceride content was measured using Triglyceride Quantification Kit (Abcam), according to manufacturer’s instructions. Tissue samples were normalized to total tissue protein concentration, measured with Protein Assay Dye Reagent Concentrate (Bio-Rad), according to manufacturer’s instructions.

#### Quantitative PCR

For gene expression analysis mice were sacrificed in *ad libitum* conditions 2 h post injection with PBS, AMPH or PEGyAMPH, tissues were collected and immediately frozen. RNA from SCG and BAT was extracted using RNeasy Plus Micro Kit (Qiagen) and from all other tissues the RNA was extracted using PureLink RNA Mini Kit (Life Technologies) according to manufacturer’s instructions. From total tissue RNA of all samples complementary DNA was reverse-transcribed by using SuperScript II (Invitrogen) and random primers (Invitrogen). Quantitative PCR was performed with SYBR Green (Invitrogen) in ABI QuantStudio 7 (Applied Biosystems). Glyceraldehyde 3-phosphate dehydrogenase (*GAPDH*) was used as housekeeping gene to normalize liver and gastrocnemius muscle tissue samples. Acidic ribosomal phosphoprotein P0 (*Arbp0*) was used as housekeeping gene to normalize adipose tissues samples. The list of primers used is shown in Table S1.

#### Histopathology Analyses

Mouse tissues were fixed in buffered formalin, and inclusion in paraffin was done according to standard technical procedures. Histopathology studies were performed on formalin-fixed and paraffin-embedded sections of 3–6 μm thick for Haematoxylin and Eosin. Tissues were analysed with a Leica DM LB2 microscope, and images captured with a Leica DFC 250 camera.

#### Intracellular Calcium-Imaging

For Ca^2+^ experiments, sympathetic neurons were obtained from *TH-cre;* CAG-LSL*-GCaMP3* mice. At 7 DIV, coverslips with sympathetic neurons from GCaMP3^+^ mice were mounted on an inverted microscope with epifluorescent optics (Axiovert 135TV, Zeiss) equipped with a xenon lamp (located at a Lambda DG-4, Sutter Instrument) and band-pass filter of 450-490 nm wavelengths. Ca^2+^ measurements were performed at 37°C, as reported in (Jacob et al., 2014). Throughout the experiments the Ach was applied focally through a drug-filled micropipette placed under visual guidance over a single neuronal cell. Drug release was performed by focal pressure (10 psi for 40 s) through a Toohey Spritzer pressure System Ile (Toohey Company). Pressure application of external physiological solution did not cause any measurable change in intracellular Ca^2+^ concentration. Images were obtained every 250 ms by exciting the preparations at 450-490 nm and the emission wavelength was set to 510 nm. Neurons were imaged with a cooled CCD camera (Photometrics CoolSNAP fx), processed and analysed by using the software MetaFluor (Molecular Devices). Ca^2+^ levels were recorded at the cell body of neurons (manually defined over the cell profile) in the field of view and variations were estimated as changes of the fluorescence signal over the baseline (ΔF/F0 = [(F_post_ – F_rest_)/F_rest_]).

#### Electrophysiology

Whole cell patch-clamp recordings performed at 7 DIV in dissociated cultures of sympathetic neurons from C57BL/6 mice using an upright microscope (Zeiss Axioskop 2FS) equipped with differential interference contrast optics by using a Zeiss AxioCam MRm camera and a x40 IR-Achroplan objective. During recordings, cells were continuously superfused with artificial cerebrospinal fluid containing (in mM: 124 NaCl, 3 KCl, 1.2 NaH_2_PO_4_, 25 NaHCO_3_, 2 CaCl_2_, 1 MgSO_4_ and 10 glucose), which was continuously gassed with 95% O_2_/5% CO_2_. Recordings were performed at room temperature in current-clamp or voltage-clamp mode [holding potential (Vh) = -60 mV] with an Axopatch 200B amplifier (Axon Instruments) (Félix-Oliveira et al., 2014). Briefly, patch pipettes with 4 to 7 MΩ resistance when filled with an internal solution [containing (in mM): 125 K-gluconate, 11 KCl, 0.1 CaCl_2_, 2 MgCl_2_, 1 EGTA, 10 HEPES, 2 MgATP, 0.3 NaGTP, and 10 phosphocreatine, pH 7.3, adjusted with 1 M NaOH, 280-290 mOsm] were used to record excitatory synaptic currents and action potential activity. The junction potential was not compensated for, and offset potentials were nulled before gigaseal formation. The resting membrane potential was measured immediately upon establishing whole cell configuration. Firing patterns of sympathetic neurons were then immediately assessed in current-clamp mode by injection of 500 ms current pulses (-25–275 pA in 12.5 or 25 pA increments) from an initial holding potential (Vh) of −70 mV. For each neuron, the threshold for action potential generation was determined by membrane potential at which phase plot slope reached 10 mV/ms (Naundorf et al., 2005). For each neuron, Δ depolarization for AP firing was calculated as the difference between the resting membrane potential and the threshold for action potential generation.

#### Fourier-Transform Ion Cyclotron Resonance (FT-ICR) Mass Spectrometry

12 weeks old male C57BL/6 mice were injected i.p. and sacrificed 30 min post-injection with AMPH or PEGyAMPH (dose: 120 μmol/kg of BW for both drugs). Brain samples were snap-frozen in liquid nitrogen before extraction procedures (Agudelo et al., 2014). Whole brain samples were smashed and extracted using ice-cold 1 mM perchloric acid (500 μL per sample) and left extracting overnight. After this time, the samples were centrifuged twice for 20 min at 5000 rpm, 4°C. Supernatants were transferred to new vials, frozen and freeze dried overnight each time, concentrated to 50 μL. Then, 25 μL of the remaining solutions were diluted in 75 μL of an electrospray ionization solution (CH_3_CN:H_2_O, 3:1). The samples were then evaluated through direct injection by using a Fourier-transform ion cyclotron resonance (FT-ICR) mass spectrometer (Bruker Apex Ultra, 7 Tesla actively shielded magnet).

#### Quantitative Liquid Chromatography with Mass Spectrometry Detection

8-12 weeks old C57BL/6 mice were injected i.p. and sacrificed 30 min post-injection with AMPH or PEGyAMPH (dose: 120 μmol/kg of BW for both drugs). Plasma and tissue samples were snap-frozen in liquid nitrogen upon collection and extraction procedures were prepared by a protein crash method for the extraction and quantitative analysis of drug content. Briefly, around 100 mg of tissue (brain and heart) were added to 100 μL of water inside a plastic screw-cap Eppendorf vial, followed by the addition of 100 μL of the stable isotope amphetamine internal standard (Amphetamine-d11 at 100 nM in water). Then a 5 mm stainless steel ball bearing was added to each sample. The samples were then homogenized using a Bioprep-24-1004 homogenizer (Allsheng) run at speed; 5 m/s, time; 30 seconds for 2 cycles. Then, 250 μL of acetone was added to each sample to precipitate any proteins in the solution. The samples were thoroughly vortexed to ensure optimal analyte recovery (recovery was >75 %). The samples were then centrifuged (5 min at ∼20,000 g) to produce a clear supernatant separate from any solid particles. The supernatant was then transferred in to a separate 2 mL amber glass auto-sampler vial (Agilent Technologies). The acetone solvent was then evaporated-off by concentrating the sample on an Eppendorf Concentrator Plus system (Eppendorf) run for 20 minutes at 60 degree Celsius. The remaining sample (∼200 μL) was then transferred into a 300 μL low-volume vial insert inside a 2 mL amber glass auto-sample vial ready for liquid chromatography with mass spectrometry detection (LC-MS). Full chromatographic separation of the analytes (AMPH and PEGyAMPH) was achieved using Shimadzu HPLC System (Shimadzu UK Limited) with the injection of 5 μL onto a Acquity UPLC® BEH C18 column; 1.7 μm, I.D. 2.1 mm X 50 mm (Waters), maintained at 40°C. Mobile phase A was water with 0.1% formic acid. Mobile phase B was acetonitrile with 0.1% formic acid. The flow was maintained at 500 μL per minute through the following gradient: 0.00 minutes_1% mobile phase B; 1.00 min, 1% mobile phase B; 2.00 min, 95% mobile phase B; 3.30 min, 95% mobile phase B; 3.40 min, 1% mobile phase B; 6.50 min, 1% mobile phase B. The sample injection needle was washed using acetonitrile with 0.1 % formic acid. The mass spectrometer used was the Exactive Orbitrap with a heated electrospray ionization source (Thermo Fisher Scientific). The mass spectrometer was calibrated immediately before sample analysis using positive and negative ionization calibration solution (recommended by Thermo Fisher Scientific). Additionally, the heated electrospray ionization source tune files were optimized for both AMPH and PEGyAMPH independently and applied to the mass spectrometry method by segmenting the MS method; this produced the lowest limit of quantitation for each compound. AMPH segment 1 was run in positive mode from 0 to 2.9 minutes with the mass spectrometer resolution set to 50,000 with a full-scan range of m/z 60 to 1200 Da. PEGyAMPH segment 2 was run in positive mode from 2.9 to 5 minutes with the mass spectrometer resolution set to 50,000 with a full-scan range of m/z 60 to 1200 Da. Analyte quantification was achieved by extracting the expected analyte masses (AMPH: 136.11208 [M+H]+ and 119.0861 [M+H-NH3]+ at retention time 2.73 minutes; AMPH-d11: 147.18112 [M+H]+ and 130.1551 [M+H-NH3]+ at retention time 2.72 minutes; PEGyAMPH: 354.22750 [M+H]+ at retention time 3.12 minutes). The area under the curve of these high resolution extracted ion chromatograms (with a window of ± 8 ppm) were normalized to the internal standard (amphetamine-d11) to account for extraction and instrument variations and then compared to a quantitative calibration line (lower limit of quantitation: 10 nM; upper limit of quantitation: 1,000 nM, for both compounds). The calculated concentrations of the analytes were then divided by the amount of tissue used in the extraction protocol to give the final results in nM per mg of tissue extracted (nM/mg).

#### Docking Calculations and Molecular Mechanics Minimizations

The X-ray structure reported for these receptors in complex with epinephrine (pdb ID: 6H7J and 4LDO, respectively - Ring et al. (2013) was used as a 3D model of the protein complex. The Docking calculations between the ligands and the receptor were performed with PatchDock Server and FireDock (Schneidman-Duhovny et al., 2005). Molecular mechanics minimizations were then carried out on the complexes using AMBER 18 package, (D. A. Case, I. Y. Ben-Shalom, S. R. Brozell, D. S. Cerutti, T. E. Cheatham, III, V. W. D. Cruzeiro, T. A. Darden, R. E. Duke, D. Ghoreishi, M. K. Gilson, H. Gohlke, A. W. Goetz, D. Greene, R Harris, N. Homeyer, Y. Huang,S. Izadi, A. Kovalenko, T. Kurtzman, T. S. Lee, S. LeGrand, P. Li, C. Lin, J. Liu, T. Luchko, R. Luo, D. J.Mermelstein, K. M. Merz, Y. Miao, G. Monard, C. Nguyen, H. Nguyen, I. Omelyan, A. Onufriev, F. Pan, R. Qi, D. R. Roe, A. Roitberg, C. Sagui, S. Schott-Verdugo, J. Shen, C. L. Simmerling, J. Smith, R. Salomon Ferrer, J. Swails, R. C. Walker, J. Wang, H. Wei, R. M. Wolf, X. Wu, L. Xiao, D. M. York and P.A. Kollman (2018), AMBER 2018, *University of California, San Francisco*), which was implemented with ff14SB (Maier et al., 2015) and GAFF (Wang et al., 2004) force fields. Parameters for the ligands (AMPH and PEGyAMPH) were generated with the antechamber module of AMBER, using GAFF force field and with partial charges set to fit the electrostatic potential generated with HF/6-31G(d) by RESP (Bayly et al., 1993). The charges were calculated according to the Merz-Singh-Kollman scheme using Gaussian 16.(Frisch, M. J.; Trucks, G. W.; Schlegel, H. B.; Scuseria, G. E.; Robb, M. A.; Cheeseman, J. R.; Scalmani, G.; Barone, V.; Petersson, G. A.; Nakatsuji, H; et al. *Gaussian 16 rev*. *B.01*, 2016, Wallingford, CT) The complexes were immersed in a water box with a 10 Å buffer of TIP3P water molecules (Jorgensen et al., 1983) and neutralized by adding explicit counter ions. A two-stage geometry optimization approach was performed with a total of 5000 minimization steps and using the default settings of AMBER 18. The first stage minimizes only the positions of solvent molecules and ions, and the second stage is an unrestrained minimization of all the atoms in the system.

### Quantification and Statistical Analysis

The number of animals used in each experimental setting (n) and the analysis performed are specified in the figure legends. The statistical analyses were performed with GraphPad Prism software (San Diego, CA) using unpaired Student’s t-test (two-tailed) or one-way ANOVA for single comparisons, with one group indicated as the control, and two-way ANOVA when comparing changes over time. p< 0.05 was considered statistically significant. Data are represented as mean ± S.E.M. Data displayed normal variance.

## Supplemental Information

Document S1. Figures S1–S7 and Table S1

Data S1. Coordinates for the Minimized Complexes of Drug-Receptor Interactions, Together with the Solutions Obtained from PatchDock-FireDock, Related to Figure 3

Document S2. Article plus Supplemental Information
