# Supplementary material for: Brain-Sparing Sympathofacilitators Mitigate Obesity without Adverse Cardiovascular Effects
Source: Cell Metab. 2020 Jun 2;31(6):1120–1135.e7. doi: 10.1016/j.cmet.2020.04.013 (PMC7671941; doi:10.1016/j.cmet.2020.04.013)
Supplement: Document S1. Figures S1–S7 and Table S1 [file mmc1.pdf]

Cell Metabolism, Volume *31*

Supplemental Information

Brain-Sparing *Sympathofacilitators* Mitigate Obesity without Adverse Cardiovascular Effects

Inês Mahú, Andreia Barateiro, Eva Rial-Pensado, Noelia Martinéz-Sánchez, Sandra H. Vaz, Pedro M.S.D. Cal, Benjamin Jenkins, Tiago Rodrigues, Carlos Cordeiro, Miguel F. Costa, Raquel Mendes, Elsa Seixas, Mafalda M.A. Pereira, Nadiya Kubasova, Vitka Gres, Imogen Morris, Carolina Temporão, Marta Olivares, Yolanda Sanz, Albert Koulman, Francisco Corzana, Ana M. Sebastião, Miguel López, Gonçalo J.L. Bernardes, and Ana I. Domingos

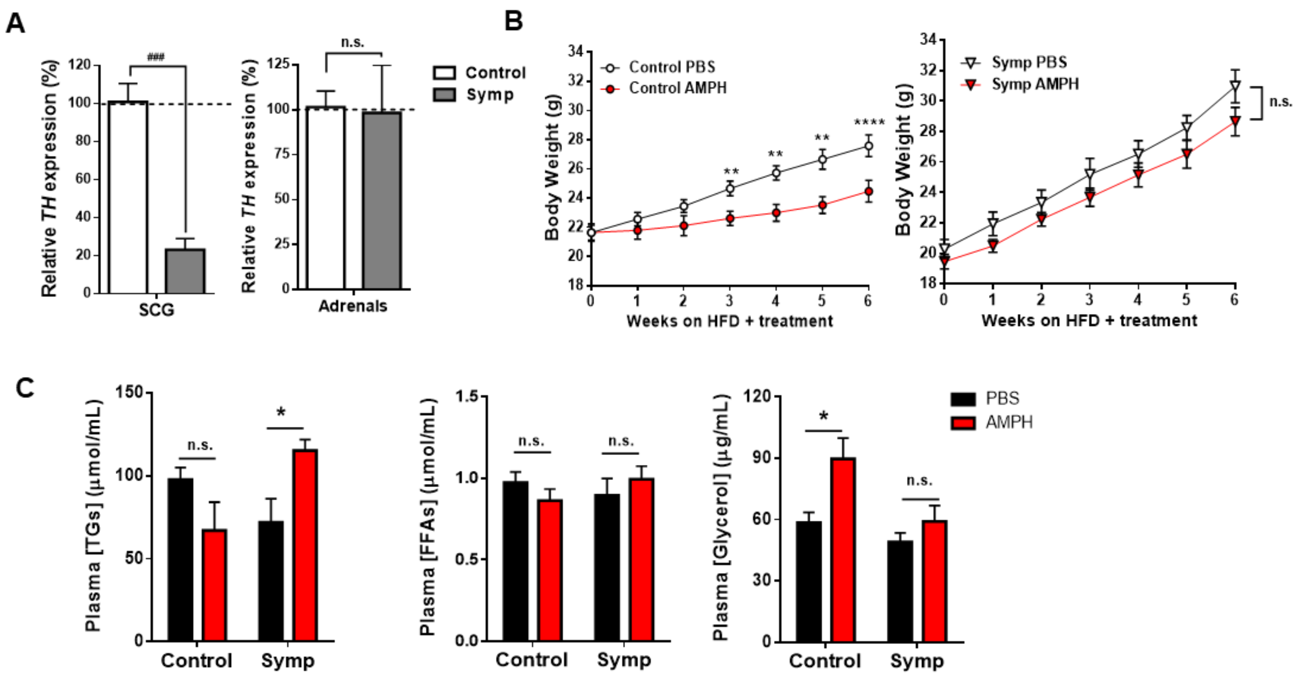

Figure S1. The *sympathomimetic* action of AMPH is required for its anti-obesity effect and the elevation of lipolysis:

A. Levels of *TH* mRNA expression, in the superior cervical ganglia (SCG) and in the adrenal glands of Control and Symp mice, determined by qRT-PCR relative to the housekeeping gene *GAPDH* (n = 8. Statistics done using unpaired Student's *t*-test, with Holm-Sidak correction method). **B.** Body weight of Control (left) and Symp (right) mice during 6 weeks of HFD exposure and treatment with PBS or AMPH (dose: 120µmol/kg of BW, daily IP injections - n = 6-12. Statistics done using two-way ANOVA). **C.** Plasma Triglycerides (TGs), Free Fatty Acids (FFAs) and Glycerol content in HFD fed Control and Symp mice 2h post-injection without access to food. (n = 6-12. Statistics done using unpaired Student's *t*-test, with Holm-Sidak correction method).

*,#p<0.05; *PBS versus AMPH, #Control versus Symp. Data presented as mean ± S.E.M. Related to Figure 1.

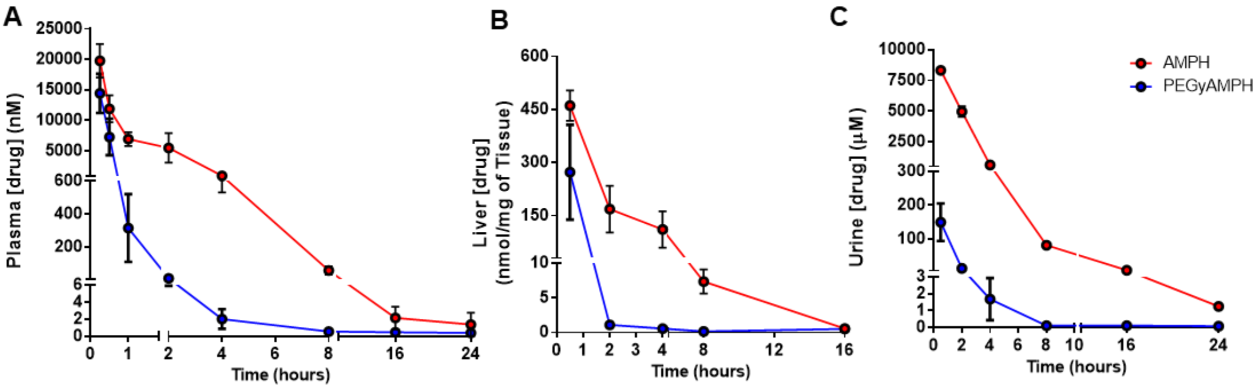

D

Slc6a2 *(h)* ([^{3}H]nisoxetine)		
**Compound I.D.**	**Concentration**	**% Control Uptake (Mean ± SEM)**
AMPH	5.0E-05 M	18.10 ± 2.10
PEGyAMPH	5.0E-05 M	98.65 ± 2.05

Figure S2. PEGylation of AMPH reduces excretion and alters its pharmacological properties:

A. Time course of the plasma concentration of AMPH or PEGyAMPH, post IV injection (dose: 120 µmol/kg of BW), assessed by mass spectrometry. **B-C.** Time course of the concentration of AMPH or PEGyAMPH, in the liver (**B**) and in the urine (**C**) of C57BL/6. **D.** Summary of the radioligand ([^{3}H]nisoxetine) competition assays with AMPH and with PEGyAMPH (0,5µM) for the *in vitro* binding to Slc6a2.

Data presented as mean ± S.E.M. Related to Figure 2.

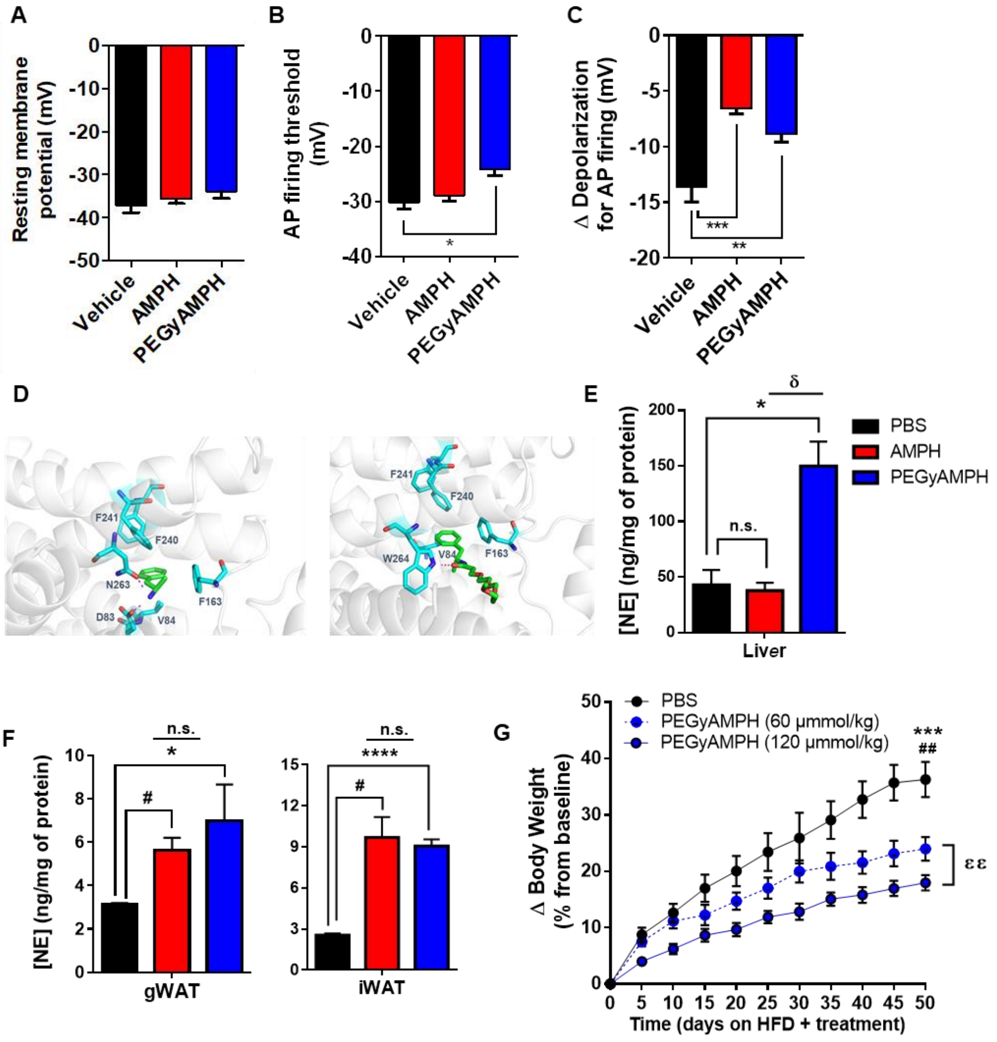

Figure S3. PEGyAMPH facilitates SNS activation and increases NE availability in target tissues, protecting mice from diet-induced obesity (DIO) in a dose-dependent manner: **A.** Resting membrane potential**. B.** AP firing threshold and **C.** Δ depolarization for AP firing of Vehicle, AMPH and PEGyAMPH-treated neurons (n = 8; Statistics done using one-way ANOVA followed by Bonferroni correction). **D.** 3D structure of β_1-adrenoceptor in complex with AMPH and PEGyAMPH. Left: Minimized structure calculated by Molecular Mechanics (MM) for the β_1-adrenoceptor/AMPH complex. Right: Minimized structure for the β_1-adrenoceptor/PEGyAMPH complex, showing the most relevant interactions the ligands and the receptor. The receptor is represented as white ribbons and the carbon atoms of the residues of the receptor interacting directly with the ligands are in blue. The carbon atoms of the ligands are in green. **E.** NE content in the Liver and **F.** NE content in gonadal and inguinal White Adipose Tissue (gWAT and iWAT, respectively) of C57BL/6 mice post-injection, with PBS, AMPH and PEGyAMPH (dose: 120 µmol/kg of BW for both drugs, IP - n = 8-10. Statistics done using unpaired Student's *t*-test, with Holm-Sidak correction.). **G.** ΔBW of C57BL/6 mice exposed to HFD and treatment with PBS or two different doses of PEGyAMPH (60 and 120 µmol/kg of BW, daily IP injections - n = 10-13. Statistics done using unpaired two-way ANOVA). $^{*,\#,\delta,\varepsilon}p<0.05$; *PBS versus PEGyAMPH; $^{\#}$PBS versus AMPH; $^{\delta}$AMPH versus PEGyAMPH; $^{\varepsilon}$60 µmol/kg-PEGyAMPH versus 120 µmol/kg -PEGyAMPH. Data presented as mean ± S.E.M. Related to Figure 3.

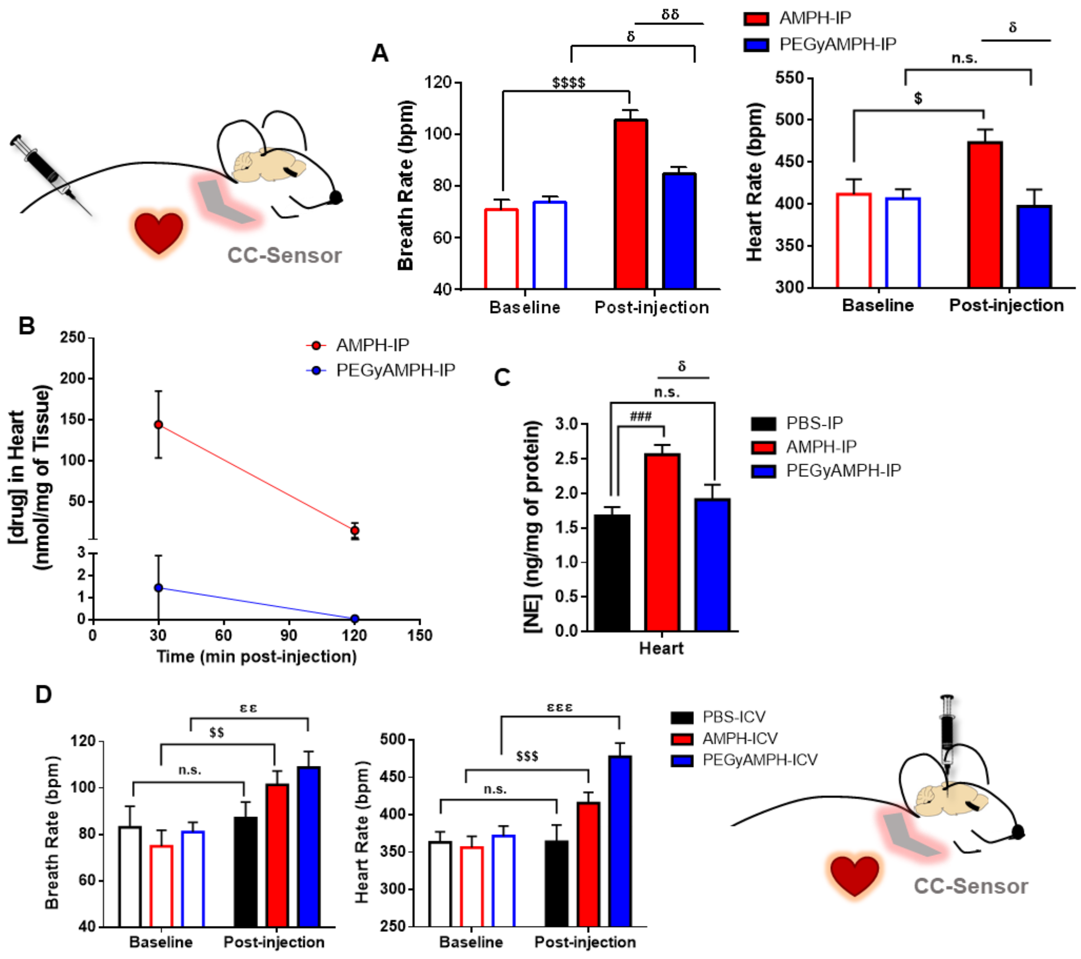

Figure S4. PEGyAMPH, unlike AMPH, does not affect cardiovascular function, unless centrally administrated:

A. Breath Rate and Heart Rate measured under anaesthesia (1-2% isoflurane) using a CC-Sensor for pulse-oximetry, before and 30-45min post IP injection with PEGyAMPH or AMPH (dose: 120 µmol/kg of BW for both drugs). **B.** Drug concentration in the Heart of C57BL/6 mice injected post IP injection, assessed by mass spectrometry. **C**. Heart NE content measured 30 min post IP injection. **D.** Breath Rate and Heart Rate measured before and 15-30min post ICV injection with either PBS, AMPH or PEGyAMPH (bolus of 60nmol, per animal) of C57BL/6 mice. (n = 8-12. Statistics done using unpaired Student's *t*-test, with Holm-Sidak correction method).

$^{\#,\$,\delta,\varepsilon}p<0.05$; $^{\#}$PBS vs AMPH; $^{\delta}$AMPH versus PEGyAMPH; $^{\$}$Baseline versus AMPH; $^{\varepsilon}$Baseline versus PEGyAMPH. Data presented as mean ± S.E.M. Related to Figure 4.

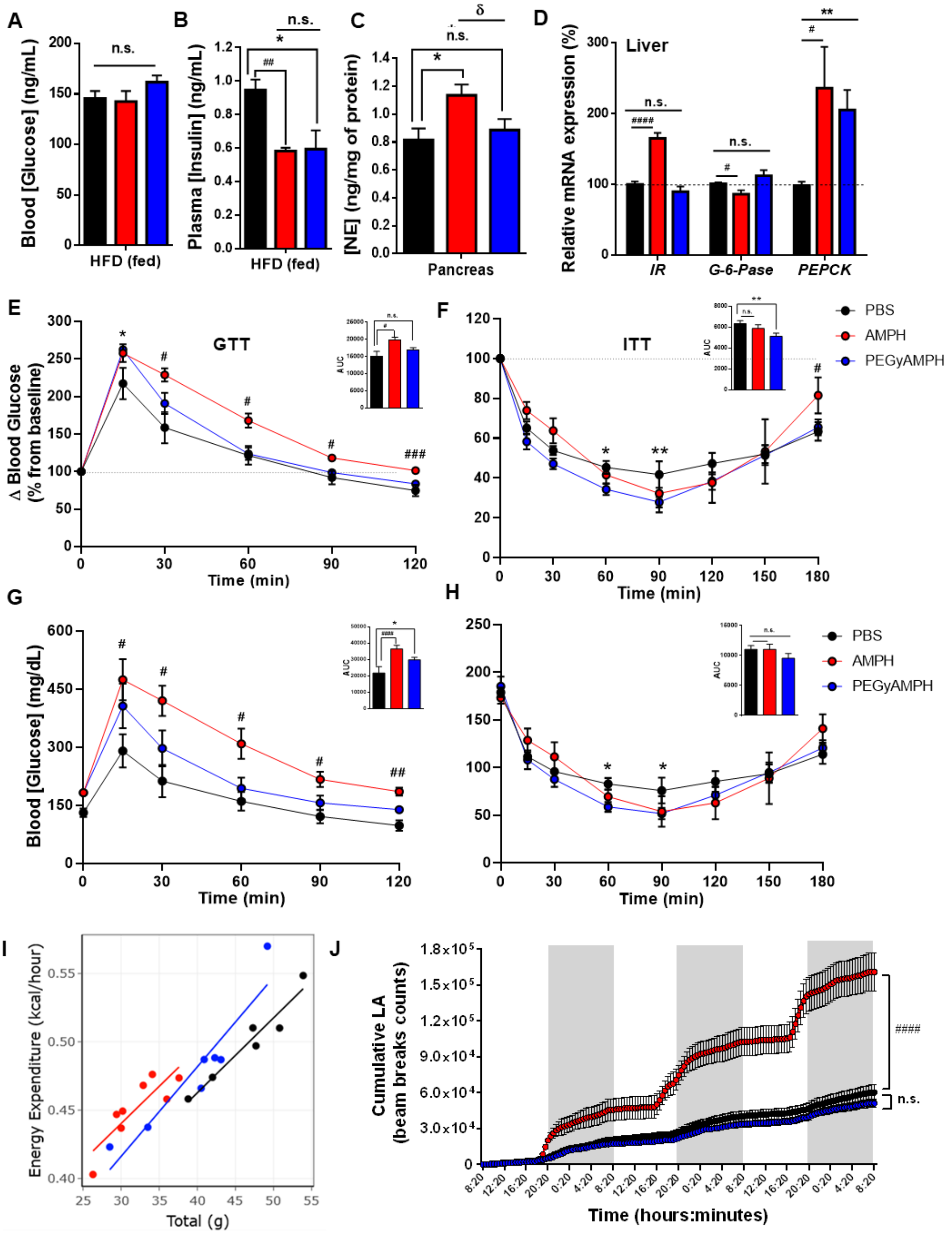

Figure S5. PEGyAMPH improves insulin sensitivity by increasing EE, without affecting Locomotor Activity (LA):

A. Blood Glucose and **B.** Plasma Insulin levels, **C.** Pancreatic NE content. **D.** Liver gene expression levels of *IR* and gluconeogenic genes Glucose 6-phosphatase (*G-6-Pase*) and Phosphoenolpyruvate carboxykinase (*PEPCK*) determined by qRT-PCR relative to housekeeping gene *GAPDH,* of fed mice 2 h post injection and without access to food, after 10 weeks of HFD exposure and respective treatment (120 µmol/kg of BW for both drugs, daily IP injections - n = 8-15. Statistics done using unpaired Student's t-test, with Holm-Sidak correction method). (**E-J**). Metabolic and behavioural tests were performed during the fourth and fifth weeks of HFD exposure and respective treatment. **E** and **G.** Intraperitoneal Glucose

Tolerance Test (GTT, glucose bolus of 2 g/kg of BW) performed 6 h post-injection with PBS, AMPH or PEGyAMPH without access to food, and the respective AUC for 120min blood glucose levels. **F** and **H.** Insulin Tolerance Test (ITT, insulin bolus of 0.9 U/kg of BW) performed 2 h post-injection with PBS, AMPH or PEGyAMPH without access to food, and the respective AUC for 180 min blood glucose levels (n = 6-8. Statistics done using two-way ANOVA). **I.** Relation between Average EE and total BW. **J.** Cumulative Locomotor Activity (LA) measured for 72h, represented in beam-break counts.

$^{*,\#;\delta}p<0.05$; *PBS versus PEGyAMPH; #PBS versus AMPH; δPEGyAMPH versus AMPH. Data presented as mean ± S.E.M. Related to Figure 5.

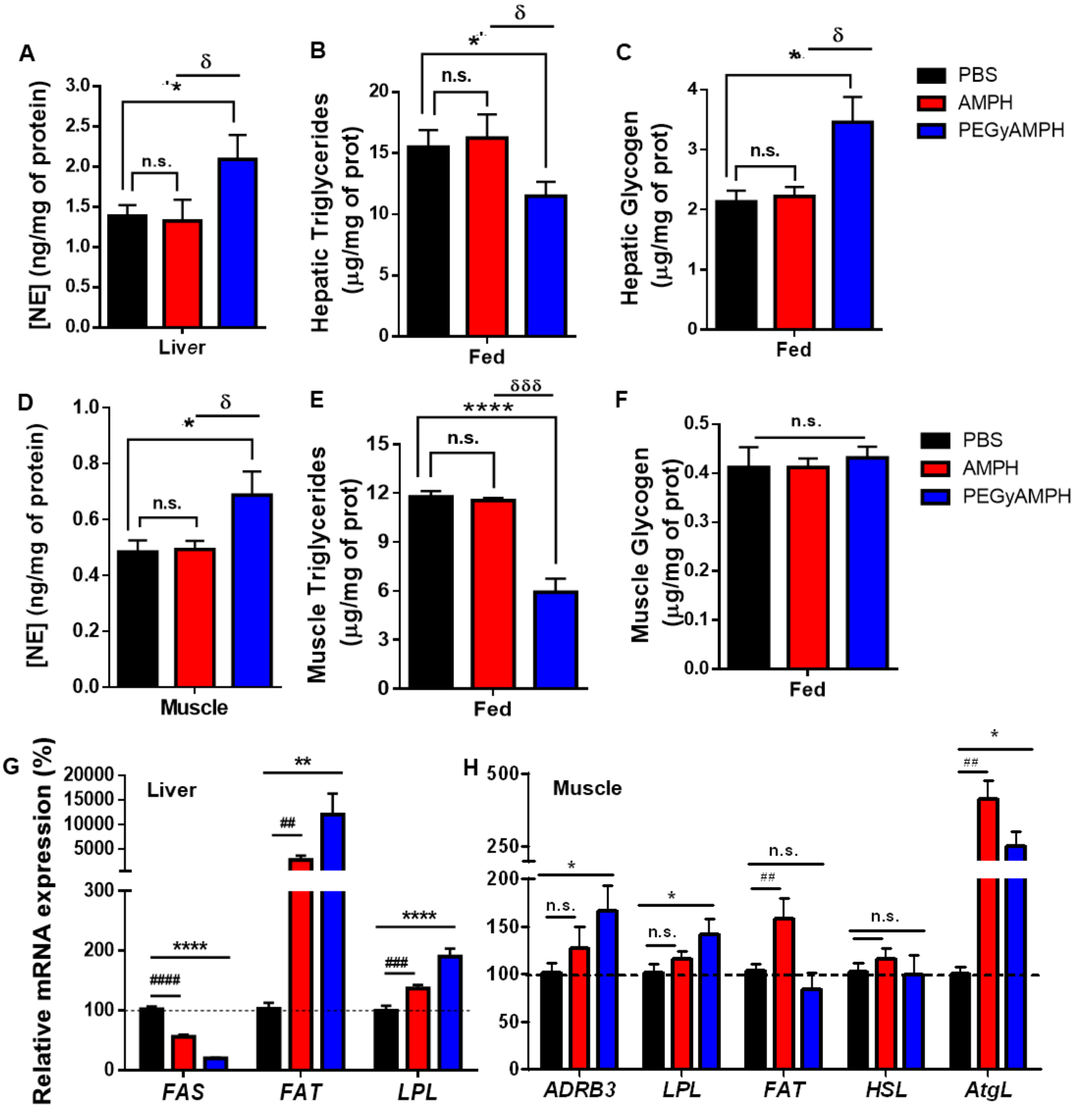

Figure S6. PEGyAMPH elevates peripheral lipid utilization during DIO:

Liver (**A-C** and **G**) and Muscle (**D-F** and **H**) measurements from C57BL/6 mice after 10 weeks of HFD exposure and chronic treatment with PBS, AMPH or PEGyAMPH. **A.** NE, **B.** TGs and **C.** Glycogen content in the liver and **D.** NE, **E.** TGs and **F.** Glycogen content in muscle, all values were normalized to total protein levels. **G.** Liver mRNA levels of Fatty Acid Transporter (*FAT*), Lipoprotein Lipase (*LPL*) and Fatty Acid Synthase (*FAS*) determined by qRT-PCR relative to housekeeping gene *GAPDH*. **H.** Muscle mRNA levels of *ADRB3*, *LPL, FAT*, *HSL* and *AtgL* determined by qRT-PCR relative to housekeeping gene *GAPDH*. (n = 12. Statistics done using unpaired Student's *t*-test, with Holm-Sidak correction.

$*,^{\delta},^{\#}p<0.05$; *PBS versuss PEGyAMPH; $^{\#}$PBS versus AMPH; $^{\delta}$PEGyAMPH versus AMPH. Data presented as mean ± S.E.M. Related to Figure 6.

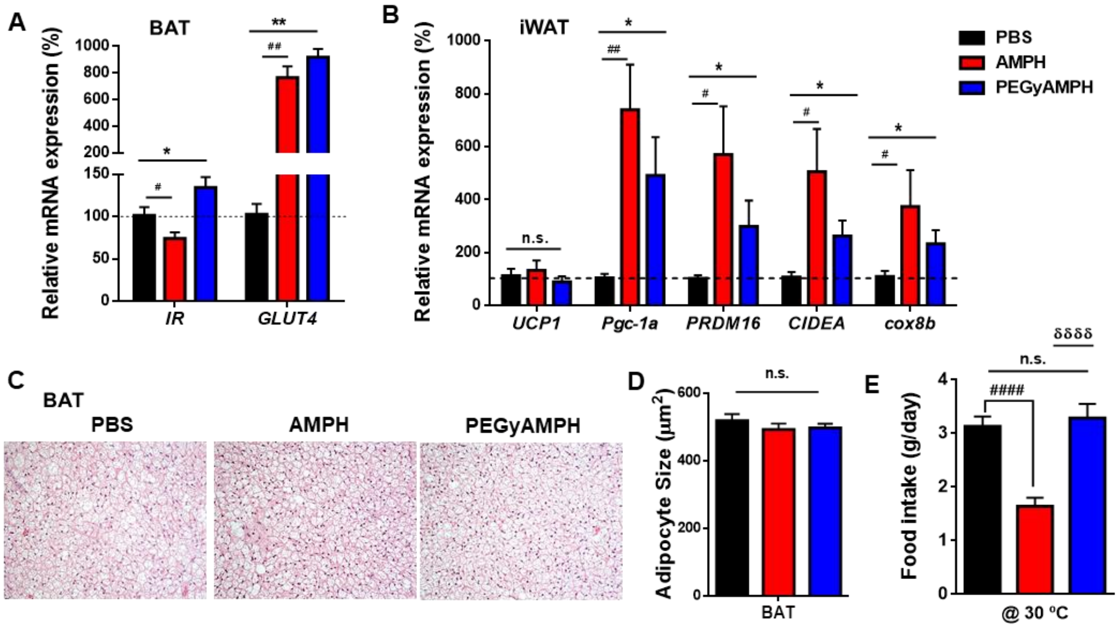

Figure S7. PEGyAMPH increases Thermogenesis during DIO:

A. BAT mRNA levels of the Insulin Receptor (*IR*) and of the Glucose Transporter type 4 isoform (*GLUT4*) and **B.** iWAT mRNA levels of thermogenic genes, gene expression was determined by determined by qRT-PCR, relative to housekeeping gene *Arbp0*. **C.** Representative Histologic Slices of BAT stained with H&E and **D.** Quantification of BAT Adipocyte Size of C57BL/6 mice after 10 weeks of HFD exposure and treatment with PBS, AMPH or PEGyAMPH (dose: 120 µmol/kg of BW for both drugs, daily IP injections). **E.** Daily Food Intake of C57BL/6 mice mice exposed to HFD and treatment with PBS, AMPH or PEGyAMPH under thermoneutral housing conditions. (n = 5-12. Statistics done using unpaired Student's *t*-test, with Holm-Sidak correction).

$^{*,\#,\delta}p<0.05$; *PBS versus PEGyAMPH; $^{\#}$PBS versus AMPH, $^{\delta}$AMPH versus PEGyAMPH. Data presented as mean ± S.E.M. Related to Figure 7.

Table S1. List of primers used for Quantitative PCR. Related to STAR Methods.

Primer	Sequence
Arbp0 Fwd	5´ CTTTGGGCATCACCACGAA 3´
Arbp0 Rev	5´ GCTGGCTCCCACCTTGTCT 3´
GAPDH Fwd	5´ AACTTTGGCATTGTGGAAGG 3´
GAPDH Rev	5´ ACACATTGGGGGTAGGAACA 3´
IR Fwd	5’ ATGGGCTTCGGGAGAGGAT 3’
IR Rev	5’ GGATGTCCATACCAGGGAC 3’
GLUT4 Fwd	5’ TTGGCTCCCTTCAGTTTGG 3’
GLUT4 Rev	5’ CTACCCAGCCACGTTGCAT 3’
G-6-Pase Fwd	5’ CGACTCGCTATCTCCAAGTGA 3’
G-6-Pase Rev	5’ GTTGAACCAGTCTCCGACCA 3’
PEPCK Fwd	5’ CTGCATAACGGTCTGGACTTC 3’
PEPCK Rev	5’ CAGCAACTGCCCGTACTCC 3’
ADRB3 Fwd	5’ ATCATGAGCCAGTGGTGGCGTGTAG 3’
ADRB3 Rev	5’ GCGATGAAAACTCCGCTGGGAACTA 3’
AtgL Fwd	5’ TGGTTCAGTAGGCCATTCCT 3’
AtgL Rev	5’ CACTTTAGCTCCAADDATGA 3’
HSL Fwd	5’ TGCTCTTCTTCGAGGGTGAT 3’
HSL Rev	5’ TCTCGTTGCGTTTGTAGTGC 3’
LPL Fwd	5’ CAGCTGGGCCTAACTTTGAG 3’
LPL Rev	5’ CCTCTCTGCAATCACACGAA 3’
FAS Fwd	5’ CCCTTGATGAAGAGGGATCA 3’
FAS Rev	5’ ACTCCACAGGTGGGAACAAG 3’
FAT/CD36 Fwd	5′ TGGCCTTACTTGGGATTGG 3′
FAT/CD36 Rev	5′ CCAGTGTATATGTAGGCTCATCCA 3′
Ucp1 Fwd	5’ ACTGCCACACCTCCAGTCATT 3’
Ucp1 Rev	5’ CTTTGCCTCACTCAGGATTGG 3’
Pgc1a Fwd	5’ CCCTGCCATTGTTAAGAC 3’
Pgc1a Rev	5’ TGCTGCTGTTCCTGTTTTC 3’
PRDM16 Fwd	5’ CAGCACGGTGAAGCCATT 3’
PRDM16 Rev	5’ GCGTGCATCCGCTTGTG 3’
CIDEA Fwd	5’ TGCTCTTCTGTATCGCCCAGT 3’
CIDEA Rev	5’ GCCGTGTTAAGGAATCTGCTG 3’
cox8b Fwd	5’ GAACCATGAAGCCAACGACT 3’
cox8b Rev	5’ GCGAAGTTCACAGTGGTTCC 3’
